# A large-scale chloroplast phylogeny of the Lamiaceae sheds new light on its subfamilial classification

**DOI:** 10.1038/srep34343

**Published:** 2016-10-17

**Authors:** Bo Li, Philip D. Cantino, Richard G. Olmstead, Gemma L. C. Bramley, Chun-Lei Xiang, Zhong-Hui Ma, Yun-Hong Tan, Dian-Xiang Zhang

**Affiliations:** 1 College of Agronomy, Jiangxi Agricultural University, Nanchang, 330045, Jiangxi, P. R. China; 2 Department of Environmental and Plant Biology, Ohio University, Athens, Ohio 45701-2979, USA; 3 Department of Biology and Burke Museum, University of Washington, Box 355325, Seattle, Washington 98195-5325, USA; 4 Herbarium, Royal Botanic Gardens Kew, Richmond, Surrey, TW9 3AE, UK; 5 Key Laboratory for Plant Diversity and Biogeography of East Asia, Kunming Institute of Botany, Chinese Academy of Sciences, Kunming 650201, Yunnan, P. R. China; 6 College of Agriculture, Guangxi University, Nanning 530004, Guangxi, P. R. China; 7 Key Laboratory of Tropical Forest Ecology, Xishuangbanna Tropical Botanical Garden, Chinese Academy of Sciences, Mengla 666303, Yunnan, P. R. China; 8 South China Botanical Garden, Chinese Academy of Sciences, Guangzhou 510650, Guangdong, P. R. China

## Abstract

Lamiaceae, the sixth largest angiosperm family, contains more than 7000 species distributed all over the world. However, although considerable progress has been made in the last two decades, its phylogenetic backbone has never been well resolved. In the present study, a large-scale phylogenetic reconstruction of Lamiaceae using chloroplast sequences was carried out with the most comprehensive sampling of the family to date (288 species in 191 genera, representing approximately 78% of the genera of Lamiaceae). Twelve strongly supported primary clades were inferred, which form the phylogenetic backbone of Lamiaceae. Six of the primary clades correspond to the current recognized subfamilies Ajugoideae, Lamioideae, Nepetoideae, Prostantheroideae, Scutellarioideae, and Symphorematoideae, and one corresponds to a portion of Viticoideae. The other five clades comprise: 1) *Acrymia* and *Cymaria*; 2) *Hymenopyramis*, *Petraeovitex*, *Peronema*, and *Garrettia*; 3) *Premna*, *Gmelina*, and *Cornutia*; 4) *Callicarpa*; and 5) *Tectona*. Based on these results, three new subfamilies—Cymarioideae, Peronematoideae, and Premnoideae—are described, and the compositions of other subfamilies are updated based on new findings from the last decade. Furthermore, our analyses revealed five strongly supported, more inclusive clades that contain subfamilies, and we give them phylogenetically defined, unranked names: Cymalamiina, Scutelamiina, Perolamiina, Viticisymphorina, and Calliprostantherina.

The circumscriptions of Lamiaceae and Verbenaceae have changed dramatically in the past 25 years as a consequence of the discovery that both families were polyphyletic as traditionally circumscribed (e.g., by Bentham^1^ and Briquet^2^ for Lamiaceae and by Briquet^3^ for Verbenaceae; see Cantino^4^ for a summary of traditional classifications of Lamiaceae). The polyphyly of Lamiaceae was first proposed based on gynoecial morphology^5^, palynology^6^,^7^, and phylogenetic analyses of non-DNA data^4^,^8^ and subsequently corroborated by molecular research^9^,^10^. Based on these studies, the traditionally circumscribed family Verbenaceae was thought to be paraphyletic (as also implied earlier by Cronquist^11^ using different terms), but more recent molecular studies of Lamiales^12^,^13^,^14^,^15^ have shown that Verbenaceae as traditionally circumscribed were polyphyletic, with genera such as *Vitex* L., *Clerodendrum* L., and *Callicarpa* L. being more closely related to the traditional Lamiaceae than they are to Verbenaceae s. str. In an attempt to delimit monophyletic families, Cantino^8^ resurrected Junell’s^5^ proposed transfer of about 50 genera (in subfamilies Caryopteridoideae, Chloanthoideae, Viticoideae, and tribe Monochileae) of Verbenaceae to Lamiaceae, leaving only subfamily Verbenoideae in the reconstituted Verbenaceae. Wagstaff *et al*.^10^ additionally found that *Congea* Roxb., a representative of subfamily Symphorematoideae of Verbenaceae, which was not transferred to Lamiaceae by Cantino^8^, should be included in Lamiaceae. Harley *et al*.^16^ adopted the expansion of Lamiaceae and proposed a subfamilial classification of the family, which is the first global, genus-level treatment of the entire family in more than a century (since Briquet^2^).

As presently circumscribed^16^, Lamiaceae are the largest family-level clade within Lamiales^17^, an order comprising 26 families and over 20,000 species^18^. They are cosmopolitan in distribution and occur as herbs, shrubs, lianas, and trees. Economically important products include teak wood (*Tectona*), oil of peppermint (*Mentha*) and patchouli (*Pogostemon*), and various culinary herbs—e.g., rosemary (*Rosmarinus*), thyme (*Thymus*), basil (*Ocimum*), oregano (*Origanum*), sage (*Salvia*), and both spearmint and peppermint (*Mentha*). Recent phylogenetic studies of angiosperms^19^, and especially Lamiales^13^,^15^, place both Lamiaceae and Verbenaceae within a large clade called “core Lamiales”^13^, where Lamiaceae are sister to a well-supported clade comprising Orobanchaceae and several small families (Mazaceae, Paulowniaceae, Phrymaceae, Rehmanniaceae), and Verbenaceae are sister to the small African family Thomandersiaceae. The early misunderstanding of the proper division between Lamiaceae and Verbenaceae relied on macroscopic features of the ovary, such as the degree to which it is divided and the placement of the style. However, what Junell^5^ recognized and Cantino^4^ later brought to the attention of botanists, is the fundamental distinction between where the ovules attach to the ovary wall relative to the false septa that divide each carpel into two single-seeded chambers; ovules in Lamiaceae attach to the sides of an inrolled carpel wall, whereas ovules in Verbenaceae attach directly to the margins of the false carpel septa. In addition, the inflorescence is fundamentally cymose in Lamiaceae versus racemose in Verbenaceae, but this is not a consistent distinction because a recemoid inflorescence has independently evolved in several subgroups of Lamiaceae. In general, Lamiaceae can be recognized by a combination of traits, including opposite leaves, bilaterally symmetric flowers with four stamens, and ovaries consisting of two fused carpels, each divided into one-seeded chambers. However, most Verbenaceae also exhibit these traits, albeit with much less variation in floral form, hence the long-standing belief that the two families are each other’s closest relatives. Only the advent of molecular phylogenetic studies^9^,^10^,^15^ showed conclusively that this was not the case.

In Lamiaceae, Harley *et al*.^16^ recognized 236 genera (comprising more than 7000 species), 226 of which were assigned to seven subfamilies: Ajugoideae, Lamioideae, Nepetoideae, Prostantheroideae, Scutellarioideae, Symphorematoideae and Viticoideae. Ten genera that could not be placed in a subfamily were listed as *incertae sedis*: *Acrymia* Prain, *Callicarpa*, *Cymaria* Benth., *Garrettia* Fletch., *Holocheila* (Kudo) S. Chow, *Hymenopyramis* Wall. ex Griff., *Ombrocharis* Hand.-Mazz., *Peronema* Jack, *Petraeovitex* Oliv., and *Tectona* L. A decade later, Harley *et al*.’s^16^ classification has been widely adopted, and new evidence has incrementally improved the classification. The monophyly of five of the seven subfamilies (Ajugoideae, Lamioideae, Nepetoideae, Prostantheroideae, and Scutellarioideae) has been supported by molecular studies^20^,^21^,^22^,^23^,^24^,^25^,^26^. Within subfamilies, intergeneric relationships have been illuminated to varying degrees in Ajugoideae^22^,^27^, Lamioideae^21^,^23^,^25^,^26^,^28^,^29^,^30^,^31^,^32^,^33^, Nepetoideae^34^,^35^,^36^,^37^,^38^,^39^,^40^,^41^,^42^,^43^, Prostantheroideae^44^,^45^,^46^, and Scutellarioideae^24^, and tribal subdivisions in Lamioideae have been proposed and updated^21^,^23^,^26^. However, the monophyly of two subfamilies, Symphorematoideae and Viticoideae, still has not been satisfactorily examined. The former is well characterized by morphological characters (e.g., woody climbing stems, capitate inflorescences surrounded by an involucre of 3–6 bracteoles, polysymmetric flowers, and ovary with incomplete septum), some of which are probably synapomorphies, but its monophyly has not been tested with DNA data because no molecular study has included more than one representative^9^,^10^,^21^,^23^,^24^,^25^,^47^. In contrast, there is now strong evidence that subfamily Viticoideae, as circumscribed by Harley *et al*.^16^, is not monophyletic. Its members fall into two clades that are not sister groups^10^,^23^,^24^,^25^,^43^,^47^, with *Vitex* (grouped with *Paravitex* H. R. Fletcher, *Petitia* Jacq., *Teijsmanniodendron* Koord., *Tsoongia* Merr., and *Viticipremna* H. J. Lam) being the largest genus in one clade and *Premna* (grouped with *Cornutia* L. and *Gmelina* L.) in the other. Based on these results, *Paravitex*, *Tsoongia*, and *Viticipremna* were reduced to synonymy with *Vitex*^47^, and *Cornutia*, *Gmelina*, and *Premna* were assigned to a provisional subfamily, “Premnoideae”^18^,^48^.

Of the ten genera considered to be *incertae sedis* by Harley *et al*.^16^, *Holocheila* has since been shown to be a member of tribe Pogostemoneae in subfamily Lamioideae^25^, and *Ombrocharis* has been shown to be part of tribe Elsholtzieae in subfamily Nepetoideae^43^. The other eight genera have still not been placed in subfamilies, but several molecular studies have shed light on their relationships: *Acrymia* and *Cymaria* form a moderately supported clade^23^,^25^,^26^; *Hymenopyramis*, *Peronema* and *Petraeovitex*^25^,^47^ or *Hymenopyramis*, *Petraeovitex* and *Garrettia*^25^ group together; *Callicarpa* is sister to the rest of the family^21^,^23^ or groups with subfamily Prostantheroideae^15^; *Tectona* emerges in various positions^21^,^23^,^47^.

Although all ten genera *incertae sedis* and all seven subfamilies have been included in molecular studies cited above, no single study has included all of them, and the phylogenetic backbone of the family remains poorly resolved. The present study employs the most broadly comprehensive sampling of the family to date, including representatives of every subfamily and tribe, all ten of the genera *incertae sedis* of Harley *et al*.^16^, and a substantially larger sample of the mainly tropical and subtropical taxa *Premna*, *Callicarpa*, *Gmelina*, *Tectona*, and Symphorematoideae than in previous studies. We are using five plastid DNA regions to infer a large-scale phylogeny of the whole family with four objectives, to: (1) increase resolution of the phylogenetic backbone of Lamiaceae, (2) determine the phylogenetic positions of the genera *incertae sedis*, (3) assess relationships among subfamilies, and (4) test the monophyly of Symphorematoideae.

## Results

The number of sequences, new sequences generated in this study, aligned length of sequences, proportion of missing data, parsimony informative characters and indels, tree length, consistency index (CI), retention index (RI), and evolutionary model, for separate and combined data sets are summarized in Table 1.

MP and ML analyses of separate data sets (*matK*, *ndhF*, *rbcL*, *rps16*, and *trnL-F*) did not yield fully resolved gene trees for the whole family. Generally, MP and ML analyses of the same data set yielded similar supported clades (Supplementary Figs S1–S5). Trees generated from different data sets had variable topological structure, but there were several comparable clades among these different trees (Table 2).

**Figure 1:**
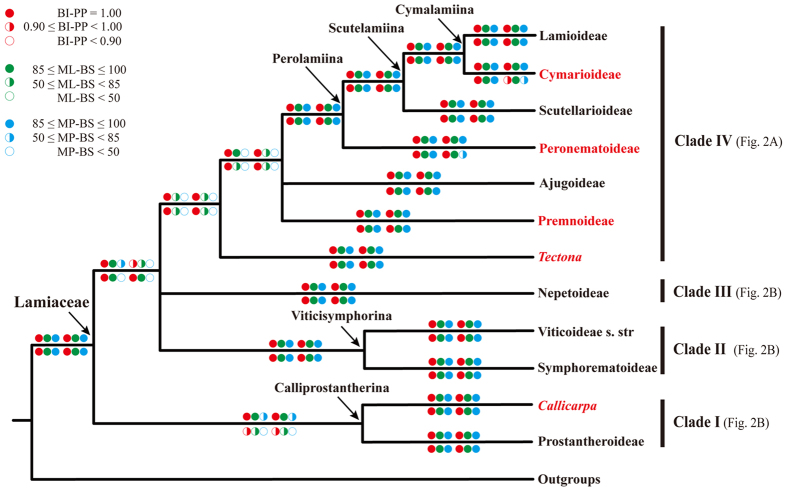
Phylogenetic backbone of Lamiaceae based on simplification of trees generated from the analyses of the combined cpDNA (*matK* + *ndhF* + *rbcL* + *rps16* + *trnL-F*) dataset D270. Color-coded circles above branches indicate support values from BI, ML and MP analyses of the combined dataset D270, with or without gaps coded respectively, while those below branches indicate support values from BI, ML and MP analyses of the combined dataset D155, with or without gaps coded respectively. Subfamilies recognized by Olmstead^46^ (Ajugoideae, Lamioideae, Nepetoideae, Prostantheroideae, Scutellarioideae, Symphorematoideae, and Viticoideae s. str.) are shown in black bold font, while new subfamilies (Cymarioideae, Peronematoideae and Premnoideae) and two genera (*Callicarpa* and *Tectona*) that we do not assign to a subfamily are in red bold font. The arrows show the clade node of Lamiaceae and the nodes of five new clades (Cymalamiina, Scutelamiina, Perolamiina, Viticisymphorina, and Calliprostantherina).

**Figure 2:**
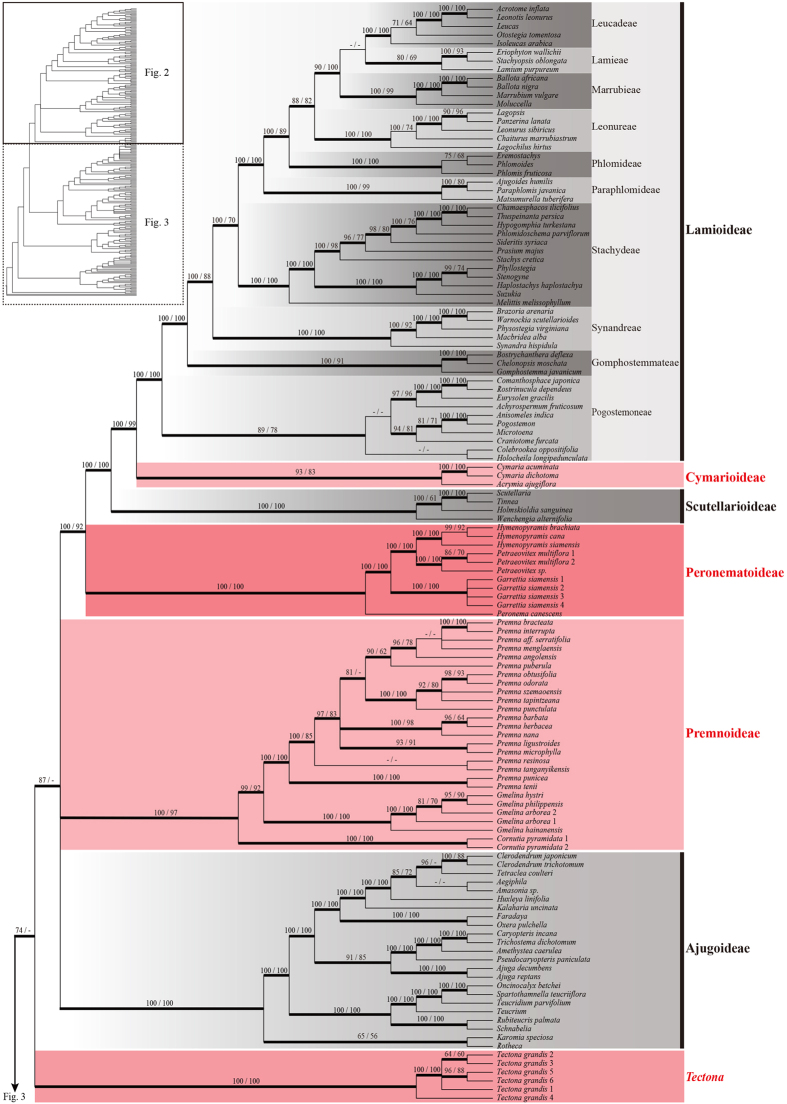
Bayesian 50% majority-rule consensus tree (box on the top left corner shows the topology) based on the combined cpDNA (matK + ndhF + rbcL + rps16 + trnL-F) dataset D270, with gaps treated as simple indels, showing the taxa from Lamioideae, Cymarioideae, Scutellarioideae, Peronematoideae, Premnoideae, Ajugoideae, and *Tectona*. The topologies of the ML and MP trees are congruent with the BI tree. Bayesian posterior probability values ≥ 0.90 are marked with bold lines. Bootstrap values ≥ 50% in ML and MP analyses are plotted above the branches, successively, while “-” indicates support values of less than 50%. Multiple accessions of the same species are numbered according to Supplementary Table S1. A single generic name indicates that the combined sequences pooled from different species of the genus. Subfamilies and tribes recognized by Olmstead^18^ were covered by gray boxes with different grey level, while new subfamilies and clades proposed in this study were covered by pink boxes and marked in red bold font.

**Figure 3:**
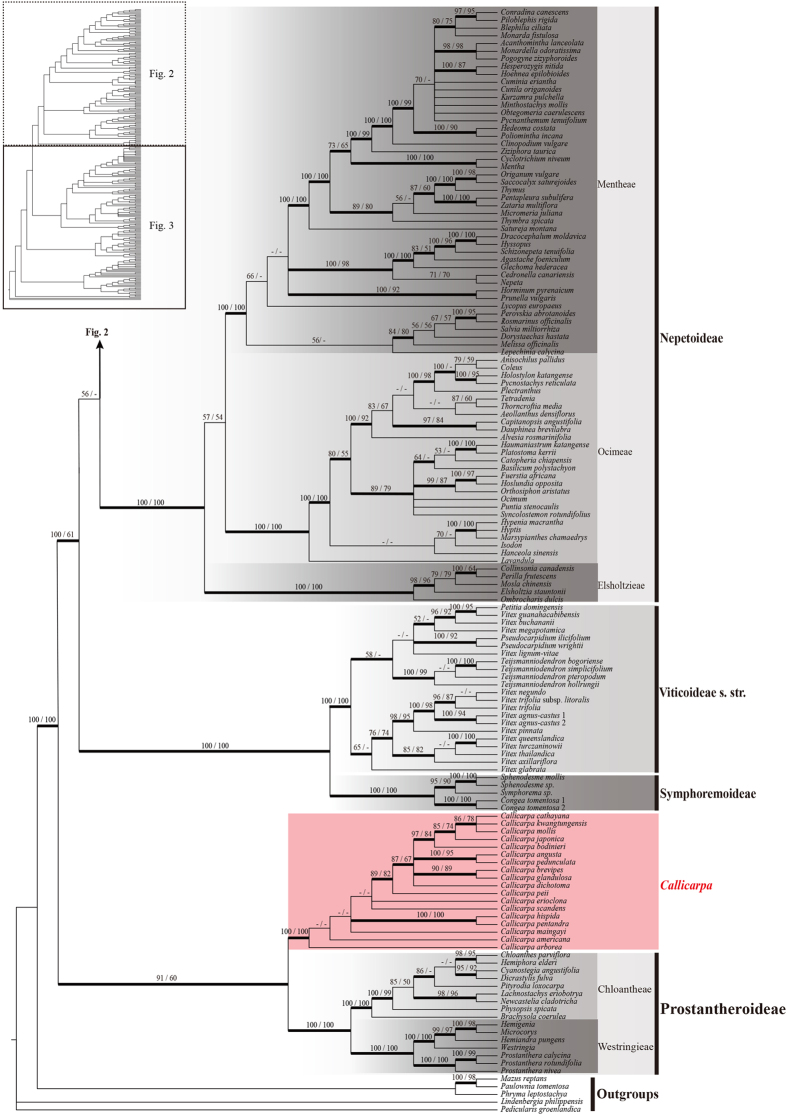
Bayesian 50% majority-rule consensus tree (box on the top left corner shows the topology) based on the combined cpDNA (matK + ndhF + rbcL + rps16 + trnL-F) dataset D270, with gaps treated as simple indels, showing the taxa from Nepetoideae, Viticoideae s. str., Symphoremoideae, *Callicarpa*, and Prostantheroideae. The topologies of the ML and MP trees are congruent with the BI tree. Bayesian posterior probability values ≥ 0.90 are marked with bold lines. Bootstrap values ≥ 50% in ML and MP analyses are plotted above the branches, successively, while “-” indicates support values of less than 50%. Multiple accessions of the same species are numbered according to Supplementary Table S1. A single generic name indicates that the combined sequences pooled from different species of the genus. Subfamilies and tribes recognized by Olmstead^18^ were covered by gray boxes with different grey level, while new subfamilies and clades proposed in this study were covered by pink boxes and marked in red bold font.

**Figure 4:**
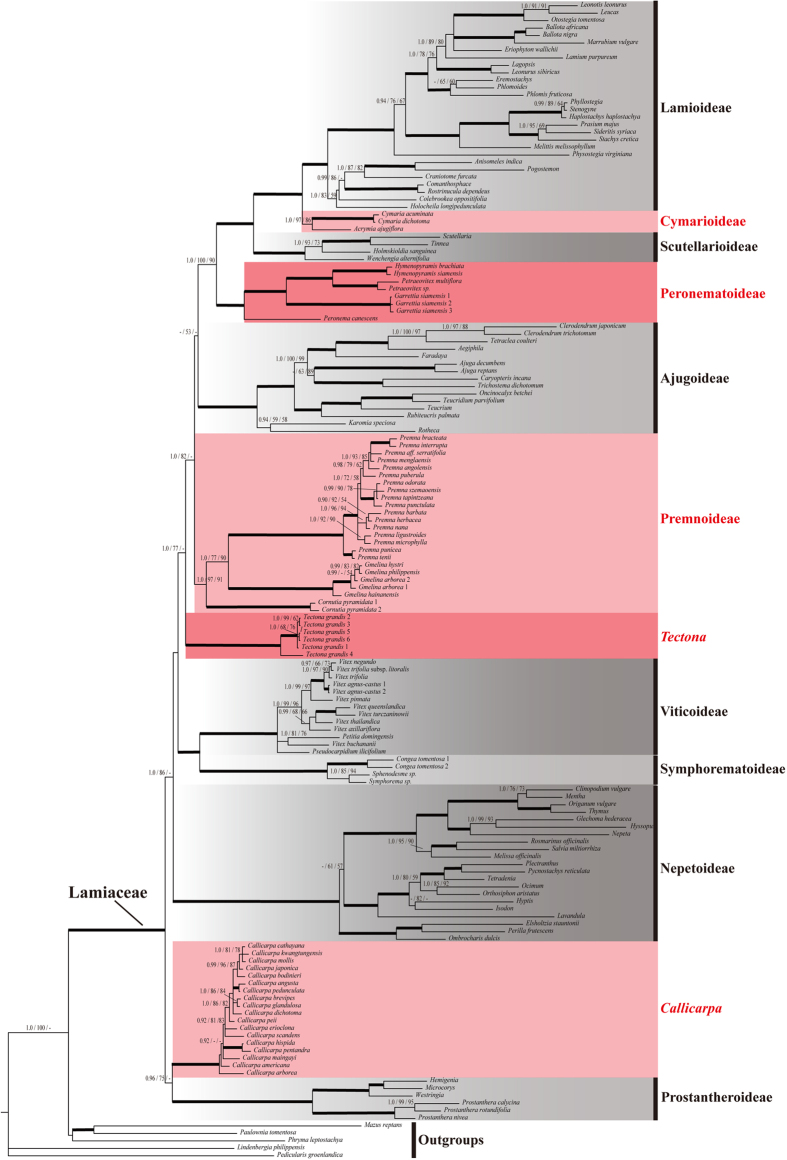
The Bayesian 50% majority-rule consensus phylogram based on combined cpDNA (*matK* + *ndhF* + *rbcL* + *rps16* + *trnL-F*) dataset D155, with gaps treated as simple indels. The topologies of the ML and MP trees are congruent with the BI tree. Support values displayed on the branches follow the order BI-PP/ML-BS/MP-BS (“−” Indicates support values of less than 0.90 in BI or 50% in ML and MP analyses, respectively). The bold lines indicate that the three support values get full scores simultaneously. Multiple accessions of the same species are numbered according to Supplementary Table S1. A single generic name represented that the combined sequences pooled from different species of the genus. Subfamilies recognized by Olmstead^45^ were covered by gray boxes with different grey level, while new subfamilies (Cymarioideae, Peronematoideae, and Premnoideae) and clades (*Callicarpa* and *Tectona*) proposed in this study were covered by pink boxes and marked in red bold font.

Based on the combined data sets D270 and D155, all MP, ML and BI analyses yielded very similar topologies, and this was true regardless of whether gaps were treated as simple indels or as missing data. Twelve well-supported primary clades were obtained in all analyses (Figs 1, 2, 3, 4; Supplementary Figs S6–S12). A simplified phylogenetic tree shows the phylogenetic backbone of Lamiaceae (Fig. 1), and the 50% majority-rule consensus tree from the BI analysis of the combined D270 data set with simple gap coding shows detailed relationships (Figs 2 and 3). Six of the 12 primary clades in Fig. 1 correspond to subfamilies Ajugoideae, Lamioideae, Nepetoideae, Prostantheroideae, Scutellarioideae, and Symphorematoideae, as recognized by Harley *et al*.^16^ and Olmstead^18^, and one corresponds to subfamily Viticoideae s. str. as recognized by Bramley *et al*.^47^. The monophyly of Symphorematoideae was confirmed for the first time, with all three genera sampled in one study (Fig. 2 and 3). Besides these subfamilial clades, the other five primary clades comprise: 1) *Acrymia* and *Cymaria*; 2) *Hymenopyramis*, *Petraeovitex*, *Garrettia*, and *Peronema*; 3) *Premna*, *Gmelina*, and *Cornutia*; 4) *Callicarpa*; and 5) *Tectona*. The relationships among these 12 clades were inferred with varying degrees of support. *Callicarpa* and Prostantheroideae group together in all of the combined-data analyses, with support ranging from low to high, and the *Callicarpa*-Prostantheroideae clade usually emerges as sister to the remaining Lamiaceae. Symphorematoideae and Viticoideae s. str. are sister groups in all combined-data analyses with high support. *Acrymia*-*Cymaria,* Scutellarioideae, and *Hymenopyramis*-*Petraeovitex*-*Garrettia*-*Peronema* are successive sister groups to Lamioideae (Fig. 2), with each node highly supported in all combined-data analyses (Fig. 1). Overall, the 12 primary clades cluster into four larger clades. Relationships among the four larger clades are poorly resolved, but each usually received moderate to high support in our analyses (Fig. 1: clade I–IV). Support values for the above-mentioned clades are summarized in Table 2. Phylogenetically defined names (names ending in *–ina* in Fig. 1) are hereby proposed for five clades that are moderately to strongly supported in our analyses and do not already have genus or subfamily names.

## Discussion

Our phylogenetic reconstruction of Lamiaceae, on the basis of the most comprehensive sampling of Lamiaceae to date, builds on prior studies using chloroplast DNA markers^9^,^10^,^21^,^22^,^23^,^24^,^25^,^27^,^28^,^31^,^32^,^33^,^34^,^35^,^36^,^37^,^38^,^39^,^40^,^41^,^43^,^45^,^46^,^47^,^49^,^50^,^51^,^52^,^53^. All of our analyses of the concatenated datasets revealed 12 highly supported primary clades (Figs 1, 2, 3, 4), which are grouped into four moderately to highly supported larger clades (Fig. 1: clade I–IV). This set of four clades has not been identified in any previous published analysis but is consistent with the unpublished results of another combined analysis of three cpDNA regions (*ycf1* + *ycf1–rps15* + *trnL-F*; B. Drew, pers. comm.). Of the 12 primary clades, five correspond to subfamilies Ajugoideae, Lamioideae, Nepetoideae, Prostantheroideae, and Scutellarioideae as recognized both by Harley *et al*.^16^ and Olmstead^18^. We have no additional findings on these subfamilies relative to previous molecular studies^21^,^22^,^23^,^24^,^25^,^26^,^43^,^46^, so we will focus our discussion on the other seven primary clades. One of these clades corresponds to Symphorematoideae recognized by Harley *et al*.^16^ and Olmstead^18^, and another corresponds to Viticoideae s. str. recognized by Bramley *et al*.^47^. These two clades are sister groups in all our combined-data analyses. The other five primary clades comprise *Acrymia*-*Cymaria*, *Hymenopyramis*-*Petraeovitex*-*Peronema*-*Garrettia*, *Premna*-*Gmelina*-*Cornutia*, *Callicarpa*, and *Tectona*. Eight genera listed as *incertae sedis* in Harley *et al*.^16^ are included in these five clades. Resolution of their phylogenetic placements makes it possible to improve the subfamilial classification of the Lamiaceae.

### Calliprostantherina

The clade comprising *Callicarpa* and Prostantheroideae, which we are naming Calliprostantherina (see Phylogenetic Nomenclature, below), emerged as sister to the remaining Lamiaceae in our phylogeny. This finding agrees with the large-scale phylogenetic analysis of Lamiidae^15^, while in other analyses, *Callicarpa*^9^,^21^,^23^ or Symphorematoideae (represented by *Congea*)^10^,^24^,^25^ was inferred to be sister to the rest of Lamiaceae. Inconsistency among published trees probably reflects taxon sampling or insufficient data, since these conditions could impact the accuracy of phylogenetic analyses^54^,^55^. Outgroups used by Scheen *et al*.^21^, Bendiksby *et al*.^23^, Li *et al*.^24^, and Chen *et al*.^25^ are distantly related to the Lamiaceae, and Prostantheroideae were not sampled by Schäferhoff *et al*.^13^. In the present study, outgroups were selected from Mazaceae, Orobanchaceae, Paulowniaceae, and Phrymaceae, which together form the sister group to Lamiaceae in recent studies of Lamiales^12^,^13^,^14^,^15^, and the ingroup was more comprehensively and densely sampled than in previous studies. Thus, there is reason to have greater confidence in our inference of a *Callicarpa*-Prostantheroideae clade that is sister to the remaining Lamiaceae than the different inferred positions of these taxa in some previous studies.

### Viticisymphorina

The clade comprising the subfamilies Symphorematoideae and Viticoideae s. str., which we are naming Viticisymphorina (see Phylogenetic Nomenclature, below), was strongly supported in all our analyses, consistent with some previous studies^23^,^47^. Each subfamily in this clade was confirmed to be monophyletic for the first time (Figs 1, 2, 3, 4, Table 2). Though Symphorematoideae is well characterized by many morphological characters, its monophyly had never been tested in previous molecular studies because only one representative had been included^9^,^10^,^21^,^23^,^24^,^25^,^47^. With all three genera included in the present study, Symphorematoideae was confirmed to be monophyletic (Figs 1, 2, 3, 4, Table 2). Viticoideae as circumscribed by Harley *et al*.^16^ have been shown to form two clades that are not sister groups^10^,^23^,^25^,^43^,^47^, with *Vitex* (grouped with *Paravitex*, *Petitia*, *Teijsmanniodendron*, *Tsoongia*, and *Viticipremna*) being the largest genus in one clade and *Premna* (grouped with *Cornutia* and *Gmelina*) the largest genus in the other. After *Premna*, *Cornutia*, and *Gmelina* were removed from Viticoideae^18^ and *Paravitex*, *Tsoongia*, and *Viticipremna* were reduced to synonymy with *Vitex*^47^, the remaining Viticoideae consist of only four genera: *Petitia*, *Pseudocarpidium* Millsp., *Teijsmanniodendron*, and *Vitex*. We included representatives of all four of these genera in a single analysis for the first time here and the monophyly of Viticoideae s. str. was strongly supported (Figs 1, 2, 3, 4, Table 2).

Symphorematoideae and Viticoideae s. str. are morphologically distinct from each other. Symphorematoideae are woody climbers with simple leaves, 3–7-flowered capitate inflorescences with accrescent bracteoles, whereas Viticoideae s. str. are generally shrubs or trees that have simple or palmately compound leaves and lack the distinctive inflorescence structure of Symphorematoideae. However, the two subfamilies are putatively connected by several anatomical structures: 1) Ovaries without a false septum are rare in other Lamiaceae, but can be found both in Symphorematoideae and Viticoideae s. str. 2) In Symphorematoideae, orthotropous and pendulous ovules are borne in the free apex of the locules^16^. Ovules in most Lamiaceae are anatropous or hemianatropous. Junell^5^ noted that the distinctive placentation in Symphorematoideae could easily be derived from the type of placentation found in many Viticoideae.

### Premnoideae

This clade, comprising *Premna*, *Gmelina* and *Cornutia*, has been partially recovered in previous molecular phylogenetic analyses^10^,^24^,^25^,^43^, in which *Premna* and *Gmelina* always grouped together. *Cornutia* was first included by Bendiksby *et al*.^23^ and revealed to be sister to a clade containing *Premna*, *Gmelina*, and *Tectona* (though the inclusion of *Tectona* in that clade conflicts with all other studies). In all of our combined analyses, the *Premna*-*Gmelina*-*Cornutia* clade was strongly supported (Table 2). This clade is part of a larger clade that also includes Ajugoideae, *Hymenopyramis*-*Petraeovitex*-*Garrettia*-*Peronema*, Scutellarioideae, *Acrymia*-*Cymaria* and Lamioideae, consistent with the findings in Chen *et al*.^25^. Though traditionally placed in Viticoideae^3^,^16^, previous studies^10^,^23^,^24^,^25^,^43^,^47^ as well as the present one have shown that the *Premna*-*Gmelina*-*Cornutia* clade is not sister to the rest of Viticoideae *sensu* Harley *et al*.^16^. Furthermore, these three genera cannot be included in any other established subfamily based on our results. In a paper intended to contrast conventional and phylogenetic nomenclature, Cantino *et al*.^56^ informally referred to the *Premna-Gmelina* clade (the position of *Cornutia* being unknown at that time) as Premnoideae under rank-based nomenclature and Premnina under phylogenetic nomenclature, and the former name was provisionally adopted by Olmstead^18^,^48^. Below we formally describe the new subfamily Premnoideae B. Li, R. G. Olmstead & P. D. Cantino.

### Peronematoideae

This clade comprises *Petraeovitex*, *Peronema*, *Hymenopyramis* and *Garrettia*. The first three of these were inferred to form a well-supported clade^25^,^47^ that is sister to the Scutellarioideae-*Acrymia*-*Cymaria*-Lamioideae clade^25^. The same sister position to Scutellarioideae-*Acrymia*-*Cymaria*-Lamioideae was found for *Garrettia*, which was first included by Bendiksby *et al*.^23^. When *Garrettia*, *Hymenopyramis*, and *Petraeovitex* were included in the same analysis, they formed a moderately supported clade^25^. In our combined analyses, the four genera form a highly supported clade that is sister to a larger clade comprising of Scutellarioideae, *Acrymia*-*Cymaria* and Lamioideae (Figs 1, 2, 3, 4, Table 2). Olmstead^18^ suggested that a new name should be provided to accommodate the *Hymenopyramis*-*Petraeovitex*-*Peronema* clade, while *Garrettia* was still listed as having uncertain subfamilial placement in his *A Synoptical Classification of the Lamiales (Version 2.4)*. Based on the present phylogeny, the clade comprising *Hymenopyramis*, *Petraeovitex*, *Garrettia*, and *Peronema* cannot be assigned to any established subfamily, thus we here propose a new subfamilial name: Peronematoideae B. Li, R. G. Olmstead & P. D. Cantino. Formal description of the new subfamily is provided below.

### Cymarioideae

This small clade comprising *Acrymia* and *Cymaria* received high support in all our analyses of combined dataset D270, as well as in BI, ML, and MP analyses of D155 with gaps coded. It was inferred to be sister to subfamily Lamioideae with strong support in all analyses (Figs 1, 2, 3, 4, Table 2). Our findings corroborate previous molecular phylogenetic analyses, where *Cymaria*^21^ or *Acrymia*-*Cymaria*^23^,^25^ was revealed to be closely related to Lamioideae. Now that the phylogenetic relationships seem to be well established, a taxonomic decision is needed whether to expand Lamioideae to include these two genera or name a new subfamily to accommodate them. The two approaches are equally consistent with the molecular phylogenetic results; i.e., both Lamioideae s. str. and a broader Lamioideae, expanded to include *Acrymia* and *Cymaria*, are well supported in our analyses and previous studies^23^,^25^. Bendiksby *et al*.^23^ and Chen *et al*.^25^ have argued that an expanded Lamioideae would be more morphologically heterogeneous and difficult to diagnose, and they therefore recommended excluding *Acrymia* and *Cymaria* from Lamioideae. Because the *Acrymia*-*Cymaria* clade was only moderately supported in their analyses, Chen *et al*.^25^ noted that if future evidence were to strongly corroborate the existence of this clade, a new subfamily could be named to accommodate them. This hypothesis is confirmed with strong confidence in our analyses (Figs 1, 2, 3, 4, Table 2); therefore, we hereby erect the new subfamily Cymarioideae B. Li, R. G. Olmstead & P. D. Cantino and formally describe it below.

## Taxonomy and Nomenclature

### An Updated Subfamilial Classification of Lamiaceae

The most recent and widely adopted classification of Lamiaceae was proposed by Harley *et al*.^16^ in the first global, genus-level treatment of the entire family in more than a century since Briquet^2^. Harley *et al*.’s landmark work includes 226 genera assigned to seven subfamilies (Ajugoideae, Lamioideae, Nepetoideae, Prostantheroideae, Scutellarioideae, Symphorematoideae, and Viticoideae), and ten genera listed as *incertae sedis* (*Acrymia*, *Callicarpa*, *Cymaria*, *Garrettia*, *Holocheila*, *Hymenopyramis*, *Ombrocharis*, *Peronema*, *Petraeovitex*, and *Tectona*). A decade later, numerous new findings have improved the classification incrementally. The results reported here provide the basis for a revised subfamilial classification. We take this opportunity to describe three new subfamilies and to update the subfamilial classification of the family incorporating new findings since Harley *et al*.^16^. For each subfamily, we provide a brief summary of its historical classification and presently understood phylogenetic position, generic and species diversity, morphology, synapomorphies, and distribution. Of the ten genera treated as *incertae sedis* by Harley *et al*.^16^, there are only two that we do not assign to a subfamily—*Callicarpa* and *Tectona*; these genera are inserted among the subfamilies in positions consistent with the phylogeny in Fig. 1.

### Prostantheroideae Luerssen

This endemic Australian subfamily includes 17 genera and ca. 300 species belonging to two major clades, Chloantheae (12 genera) and Westringieae (5 genera). Prior to the cladistic study of Cantino^4^, these tribes were usually placed in Verbenaceae (or Chloanthaceae^57^,^58^) and Lamiaceae, respectively (e.g., Briquet^2^,^3^). However, based on gynoecial anatomy, Junell^5^ transferred “Chloanthoideae” (i.e., Chloantheae) to Lamiaceae and suggested that it shares a common origin with “Prostantheroideae” (i.e., Westringieae). Cantino *et al*.^59^ first placed the two groups together in a subfamily (“Chloanthoideae”). Monophyly of each tribe (excluding *Spartothamnella* Briq. and *Tectona* from Chloantheae, contrary to Munir^60^ and Cantino *et al*.^59^, respectively) and of the combined Prostantheroideae was confirmed by molecular phylogenetic analysis^20^, which also produced the first evidence that *Callicarpa* is sister to Prostantheroideae. Phylogenetic studies of Prostantheroideae^20^,^44^,^45^,^46^ (as well as from T. Wilson and B. Conn, pers. comm.) have revealed that several genera are not monophyletic as currently circumscribed. Recent and ongoing studies have led to abandonment of the genera *Wrixonia* F. Muell. (included in *Prostanthera* Labill.)^61^ and *Mallophora* Endl. (included in *Dicrastylis* J. Drumm. ex W. H. Harvey)^62^, the reinstatement of *Dasymalla* Endl. and *Quoya* Gaudich. and addition of *Muniria* N. Streiber & B. J. Conn comprising species previously assigned to *Pityrodia* R. Br.^63^, and indications that additional realignments to several genera will be forthcoming^44^,^64^ (also T. Wilson, pers. comm.). A probable synapomorphy is a dry schizocarp that splits into four one-seeded mericarps. This feature also characterizes several other clades within Lamiaceae, but our results indicate that it evolved independently in each. Because this fruit type is found in all members of Westringieae and in *Brachysola* Rye, which is sister to the rest of Chloantheae^4^,^20^,^45^, it is the most parsimonious assignment to the most recent common ancestor of Prostantheroideae.

### *Callicarpa* Linnaeus

*Callicarpa* contains about 140 species occurring in both temperate and tropical regions^16^. The plants are small trees or shrubs with actinomorphic, 4–5 (−7)-parted flowers and drupaceous fruits. Despite being one of the largest genera in Lamiaceae, its phylogenetic position had not previously been confirmed. In previous molecular studies, *Callicarpa* was included with only one or few representatives, and has been inferred to be sister to the rest of the family^9^,^21^,^23^, to group with subfamily Prostantheroideae^15^,^20^,^43^, or to be variably isolated in different positions^10^. Bramley^49^ sampled more representatives and indicated that *Callicarpa* is monophyletic, but she could not infer its phylogenetic position because of poor sampling from the whole family. In the present study, *Callicarpa* was sampled much more extensively (18 spp.), taking into consideration its morphological and geographic breadth and its infrageneric classification. In all our analyses, the monophyly of *Callicarpa* was well supported (Figs 1, 2, 3, 4; Table 2). A sister relationship between *Callicarpa* and subfamily Prostantheroideae was moderately to highly supported in analyses of the combined dataset D270 (Figs 1, 2, 3, Table 2), as well as in BI and ML analyses of the combined dataset D155 (Fig. 4).

### Symphorematoideae Briquet

The subfamily has three genera: *Congea* (ca. 7 species), *Sphenodesme* Jack (ca. 14 species) and *Symphorema* Roxb. (3 species). All genera are endemic to continental Asia (India to Indochina and southern and eastern China) and parts of Malesia (Peninsular Malaysia, Sumatra, Borneo, Java, Tanimbar Islands, and the Philippines). *Congea tomentosa* Roxb. and to a lesser extent *C. griffithiana* Munir are cultivated as ornamental climbers. There are several morphological traits that unite the three genera. All are climbers with inflorescences of 3–7-flowered capitate cymes. These are usually surrounded by conspicuous bracteoles, often coloured and accrescent. Flowers of *Sphenodesme* are 5 or 6-merous; the flowers of *Symphorema* are 6 to 16 (–18)-merous. The corolla of *Congea* is 2-lipped, but the corollas of *Sphenodesme* and *Symphorema* are actinomorphic. The ovaries are incompletely 2-locular, and the ovules are orthotropous and pendulous. Although unique to this subfamily, the ovary type was interpreted by Junell^5^ as being derived from that found in genera of “Viticoideae” (in which Junell included not only Viticoideae s. str. but also *Callicarpa*, *Tectona*, Premnoideae, Peronematoideae, Cymarioideae, and Ajugoideae in our classification). The fruit in all three genera is indehiscent, weakly drupaceous or dry, and is 1 (−2) -seeded by abortion.

### Viticoideae Briquet

Viticoideae, as circumscribed here, includes only three genera: *Vitex* (ca. 250 spp.), *Teijsmanniodendron* (23 spp.), and *Pseudocarpidium* (9 spp.). In contrast, Viticoideae sensu Harley *et al*.^16^ included ten genera (viz., *Petitia*, *Cornutia*, *Premna*, *Viticipremna*, *Tsoongia*, *Paravitex*, *Vitex*, *Teijsmanniodendron*, *Gmelina*, and *Pseudocarpidium*). Molecular studies, initially by Wagstaff and Olmstead^10^, had identified two distinct clades, one centered on *Vitex* and another including *Gmelina, Cornutia,* and *Premna*. Phytochemical studies^65^ hinted at the same relationships, finding that phenolic compounds present in *Premna* or *Gmelina* were absent in *Vitex*, *Petitia*, and *Teijsmanniodendron*. Because several smaller viticoid genera were not included in these early analyses, the circumscription of the subfamily was not altered by Harley *et al*.^16^. Bramley *et al*.^47^ further elucidated the relationships among the viticoid genera, focusing particularly on Southeast Asian taxa traditionally allied to *Vitex*. Based on their results, *Viticipremna*, *Tsoongia*, and *Paravitex* were included in *Vitex*, reducing the number of viticoid genera to seven. The generic status of *Petitia* and *Pseudocarpidium* was unchanged, because of poor support for the position of the former, lack of any data for the latter, and poor sampling among Neotropical taxa. In the present study, we find sufficient evidence to include *Petitia*, but not *Pseudocarpidium*, in *Vitex*. Similarly, the generic status of *Teijsmanniodendron* remains problematic. Neither the analyses of Bramley *et al*.^47^ nor our analyses provide convincing support to include *Teijsmanniodendron* in *Vitex*. Identification of species in these two genera is often confused; the sole morphological character that can be used to delimit them in most cases is a swelling present at the base and apex of the petiole in *Teijsmanniodendron*. Traditionally, *Teijsmanniodendron* species were also recognised by their capsule-like rather than drupaceous fruit that is 1-seeded (by abortion)^66^, but a reduction in the number of mature seeds can also occur in *Vitex* species.

Viticoideae are distributed predominantly in the Tropics (*Vitex* throughout; *Teijsmanniodendron* in Malesia; *Pseudocarpidium* in the Caribbean), although there are a few temperate species of *Vitex*. Madagascar may be home to a number of currently unrecognized species of *Vitex*^67^. Analysis of a greater number of viticoid taxa could result in further changes to generic boundaries in this subfamily. Possible synapomorphies for the subfamily are phytochemical (see Pedersen^65^). There has been no comparative study of morphological or anatomical characters including all of the viticoid genera aside from Junell’s^5^ work on gynoecial structure. Although Junell noted that *Vitex*, *Petitia*, *Pseudocarpidium*, and the other genera now recognized as *Vitex* have a very similar ovary structure, he found some differences in *Teijsmanniodendron*. Further comparative studies including subfamily Viticoideae, particularly focusing on gynoecial structure, may elucidate unifying characters.

### Nepetoideae (Dumortier) Luerssen

Nepetoideae is the largest subfamily of Lamiaceae, containing almost half of the genera and species. It now contains 118 genera (compared to 105 recognized by Harley *et al*.^16^) and ca. 3400 species, which are widely distributed across tropical and temperate regions of the northern and southern hemispheres but with few native species in Australia and New Zealand. Probable synapomorphies for Nepetoideae include hexacolpate and three-celled pollen, investing embryos, myxocarpy, gynobasic style, and the presence of rosmarinic acid^4^,^16^,^68^,^69^,^70^,^71^. Three tribes are now recognized within Nepetoideae^16^: Elsholtzieae, Mentheae, and Ocimeae. The monophyly of each of these tribes is well supported by molecular phylogenetic studies^34^,^37^,^40^,^43^,^72^, but there are conflicting findings about relationships among the three tribes.

There have been several genus-level changes since the treatment of the subfamily by Harley *et al*.^16^. Bräuchler *et al*.^72^ described a new genus *Killickia* Bräuchler, Heubl & Doroszenko from South Africa. Harley and Pastore^73^ did a major genus-level revision of Hyptidinae, recognizing 12 genera that were not recognized by Harley *et al*.^16^. Nine of them were new (*Cantinoa* Harley & J. F. B. Pastore, *Cyanocephalus* (Pohl ex Benth.) Harley & J. F. B. Pastore, *Eplingiella* Harley & J. F. B. Pastore, *Gymneia* (Benth.) Harley & J. F. B. Pastore, *Leptohyptis* Harley & J. F. B. Pastore, *Martianthus* Harley & J. F. B. Pastore, *Medusantha* Harley & J. F. B. Pastore, *Oocephalus* (Benth.) Harley & J. F. B. Pastore, and *Physominthe* Harley & J. F. B. Pastore), and the other three were resurrected (*Condea* Adans., *Eriopidion* Harley, and *Mesosphaerum* P. Browne). Drew *et al*.^42^ synonymized *Chaunostoma* Donn. Sm. and *Neoeplingia* Ramam., Hiriart & Medrano with *Lepechinia* Willd. Chen *et al*.^43^ resurrected *Keiskea* Miq. (included in *Collinsonia* L. by Harley *et al*.^16^) and showed that *Ombrocharis* (unassigned to subfamily by Harley *et al*.^16^) is sister to *Perillula* Maxim. within tribe Elsholtzieae. Drew and Sytsma^37^ found *Heterolamium* C. Y. Wu to be nested within *Meehania* Britton, but Deng *et al*.^74^ found that the specimen of *Heterolamium* studied by Drew and Sytsma^41^ was misidentified and was in fact a member of *Meehania*. Thus, the systematic position of *Heterolamium* within Nepetoideae is still uncertain.

### *Tectona* Linnaeus f

*Tectona* is a genus of large trees comprising three species distributed from India to southeast Asia. The large drupaceous fruits contain a hard four-celled endocarp and are enclosed in an enlarged persistent calyx. *Tectona* has been included in several molecular studies^9^,^10^,^21^,^23^,^47^, but its phylogenetic position has never been determined definitively. It has been inferred to be sister to a clade comprising *Hymenopyramis*-*Petraeovitex*-*Peronema* and *Premna*-*Gmelina*^47^, to a large clade containing Ajugoideae, Lamioideae, Scutellarioideae, *Peronema*-*Petraeovitex*, and *Premna*-*Gmelina*-*Cornutia* (B. Drew, pers. comm.), to another larger clade comprising Ajugoideae, Lamioideae, *Premna*-*Gmelina*, Prostantheroideae, Scutellarioideae, and *Vitex*-*Petitia*^10^, or to group with *Gmelina*^21^,^23^. In our more comprehensive analyses, *Tectona* is inferred to be sister to a large clade comprised of Lamioideae, *Acrymia*-*Cymaria*, Scutellarioideae, *Hymenopyramis*-*Petraeovitex*-*Garrettia*-*Peronema*, Ajugoideae, and *Premna*-*Gmelina*-*Cornutia*, with moderate to strong support in BI and ML analyses of both datasets D270 and D155, with or without gaps coded (Figs 1, 2, 3, 4). The distinct morphology of *Tectona* including an actinomorphic 5–7-lobed calyx and corolla, greatly enlarged and inflated persistent calyx, and 4-celled endocarp with small central cavity between the cells^16^, contributed to the difficulty of placing it in previous classifications. Our results suggest that *Tectona* is an early diverging lineage from the major clade IV (Figs 1, 2, 3, 4).

### Premnoideae B. Li, R. G. Olmstead & P. D. Cantino, subfam. nov

Type: *Premna* L. in Mant. ii, 154. 1771.

Trees, shrubs, lianas, or rarely small herbs. Leaves simple, opposite, usually aromatic. Inflorescence cymose, usually terminal, variable in form. Calyx tubular or campanulate, truncate or 4–5-toothed, often obscurely 2-lipped. Corolla blue, purple-violet, mauve, yellow, brownish or white, infundibular or hypocrateriform, 4–5-lobed, ±2-lipped or occasionally actinomorphic. Stamens 4 or posterior pair reduced to staminodes, didynamous or equal, included or slightly exserted, thecae separate, parallel to widely divergent; pollen usually tricolpate (4–5-colpate in *Cornutia*), tectate-perforate, psilate or suprareticulate. Ovary unlobed, stigma 2-lobed, equal or unequal; disc well developed (*Cornutia*) or absent (*Premna*, *Gmelina*). Fruit drupaceous, exocarp fleshy, pyrene hard, 4-seeded (sometimes3–1-seeded by abortion).

This new subfamily contains three genera: *Premna* (50–200 spp. in tropical to subtropical Asia, Africa, Australia, and the Pacific Islands), *Gmelina* (31 spp. in tropical and subtropical Asia to Australia and western Pacific Islands), and *Cornutia* (12 spp. in tropical America). The three genera were traditionally placed in subfamily Viticoideae of Verbenaceae^3^, and transferred to Lamiaceae together with the subfamily^5^,^16^,^59^, and then excluded from Viticoideae by Olmstead^18^ based on molecular evidence that Viticoideae is non-monophyletic if they are included^23^,^47^. A possible synapomorphy for Premnoideae is a drupaceous fruit with one four-seeded pyrene. However since a similar fruit structure is also found in *Tectona* and some species of *Vitex*, it may instead be a synapomorphy at a deeper level in the phylogeny with subsequent reversals. With the number of species estimated from 50^75^ to 200^76^, *Premna* now ranks among the most taxonomically difficult and complicated genera of Lamiaceae. Though some regional revisions of the genus have been done in recent decades^77^,^78^,^79^,^80^,^81^,^82^, there is no treatment of the genus throughout its range. A global taxonomic revision of *Gmelina* was published by de Kok^83^.

### Ajugoideae Kosteletzky

Ajugoideae contains 26 genera and ca. 760 species and is cosmopolitan in distribution. A series of phylogenetic studies, which collectively included every genus except *Monochilus* Fisch. & C. A. Mey., have resolved most of the generic boundaries and relationships^22^,^27^,^84^,^85^,^86^,^87^,^88^. Our results find a small clade comprising *Karomia* Dop and *Rotheca* Raf. to be sister to the rest of the subfamily. Unpublished results by one of us (C. L. Xiang) indicate that *Discretitheca* P. D. Cantino and *Glossocarya* Wall. ex Griff. are close relatives of *Rotheca*. This clade of four genera is distributed from Africa to the Indian subcontinent, Southeast Asia, and Queensland. The rest of Ajugoideae comprises two large clades. One, with ca. 260 species, is primarily temperate and centered on *Teucrium* L. (this clade remains poorly studied). The other large clade, with ca. 425 species, is centered on *Clerodendrum*^19^,^24^,^51^,^87^. The latter clade comprises a primarily tropical clade, which includes *Clerodendrum* and related genera, and a primarily temperate clade, which includes *Ajuga* L., *Trichostema* L., *Caryopteris* Bunge, and related genera. Molecular analyses^22^,^27^,^89^ have also increased the number of genera accepted from 24^16^ to 26, with *Huxleya* Ewart now included in *Clerodendrum*, *Faradaya* F. Muell. included in *Oxera* Labill., and four genera (*Kalaharia* Baill., *Ovieda* L., *Tetraclea* A. Gray, and *Volkameria* L.) segregated from *Clerodendrum*. Probable synapomorphies of Ajugoideae include pollen exine with supratectal spines, spinules or verrucae, and exine with branched to granular columellae. These character states are widespread in Ajugoideae^16^,^90^,^91^ and rare (branched columellae) or absent (spinules and verrucae) elsewhere in the Lamiaceae.

### Peronematoideae B. Li, R. G. Olmstead & P. D. Cantino, subfam. nov

Type: *Peronema* Jack in Malayan Misc. 2 (7): 18. 1822.

Shrubs, trees, and lianas. Leaves opposite, petiolate, simple or ternately, biternately, or pinnately compound. Inflorescence cymose, axillary, and/or terminal, highly variable in form. Calyx actinomorphic, 4–5-lobed, and usually accrescent (not accrescent in *Peronema*) in fruit. Corolla white to yellow, 4–5-lobed, and nearly actinomorphic to zygomorphic. Stamens 4 or posterior pair reduced to staminodes (*Peronema*), equal or didynamous, included or exserted, thecae parallel to divaricate, usually separate (confluent in *Garrettia*) at dehiscence; pollen tricolpate, tectate-perforate. Ovary unlobed, stigma 2-lobed, equal or unequal; disc absent or poorly developed. Fruit dry, globose or turbinate, glabrous or pubescent to villous, indehiscent or breaking into two or four mericarps, abscission-scar as long as the mericarp.

This new subfamily comprises four small, mostly tropical Asian genera that were treated as *incertae sedis* by Harley *et al*.^16^: *Garrettia* (1 sp., southwest China, Thailand, and Indonesia), *Hymenopyramis* (7 spp., India, China, and Indo-China), *Peronema* (1 sp., Thailand to Malaysia and western Indonesia), and *Petraeovitex* (8 spp., Burma, Thailand, Malaysia, Indonesia, Philippines, New Guinea, and Melanesia). Previously, *Hymenopyramis*, *Peronema*, and *Petraeovitex* have been placed in Caryopteridoideae^3^ or transferred to Viticoideae^5^, or *Hymenopyramis* was retained in Viticoideae but *Peronema* and *Petraeovitex* were transferred to Teucrioideae^59^. *Garrettia* was always placed in Caryopteridoideae^92^,^93^ before being transferred to Ajugoideae^59^. The four genera of Peronematoideae differ greatly in morphology and have never been linked in any previous classification, but Chen *et al*.^25^ has found some traits in common, including woody stems (small or climbing shrubs, lianas or large trees), white to yellowish corolla, unlobed ovary, nectar disc poorly developed or absent, and dry fruit. Each of these traits is probably either synapomorphic at a more inclusive level within Lamiaceae or plesiomorphic in the family as a whole^25^.

### Scutellarioideae (Dumortier) Caruel

A taxon centered on *Scutellaria* was recognized as a distinct element within Lamiaceae in early classifications (e.g., Bentham^1^; Briquet^2^), often comprising only *Scutellaria* and the segregate genera, *Perilomia* Kunth and *Salazaria* Torr., now included within *Scutellaria*^16^,^94^. Early phylogenetic studies based on morphology^4^,^8^ and DNA sequences^10^ expanded this clade to include *Renschia* Vatke, *Tinnea* Kotschy ex Hook. f., and *Holmskioldia* Retz., the latter formerly assigned to Verbenaceae. The rediscovery of the extremely rare *Wenchengia* C. Y. Wu & S. Chow permitted Li *et al*.^24^ to confirm its placement in Scutellarioideae by Harley *et al*.^16^. A characteristic two lobed, untoothed calyx is shared by a clade of *Scutellaria*, *Renschia*, and *Tinnea*. *Holmskioldia* is sister to this clade and has an expanded saucer-shaped calyx with five, often indistinct, lobes, which form the dominant part of the floral display, unlike the other genera. *Wenchengia* is sister to the rest of the clade and has a two-lobed, but five-toothed calyx. Probable synapomorphies for Scutellarioideae include pericarps with tuberculate or elongate processes^24^, high densities of xylem fibers in the calyces^95^, and possibly racemose inflorescences (but they are cymose in *Holmskioldia* and most species of *Tinnea*, suggesting that independent origin of racemes within Scutellarioideae may be equally parsimonious). *Scutellaria* includes approximately 360 species, is cosmopolitan in distribution, occurs in a wide range of habitats, and includes annual and perennial herbs and shrubs. A global taxonomic revision of *Scutellaria* established infrageneric classification and reduced *Harlanlewisia*, *Perilomia*, and *Salazaria* to synonymy^94^. *Tinnea* includes 19 species, all endemic to Africa. The rest of the clade consists of small, narrowly endemic genera: *Renschia* (1–2 spp., Somalia), *Holmskioldia* (1 sp., southern Himalayas), and *Wenchengia* (1 sp., Hainan island, China).

### Cymarioideae B. Li, R. G. Olmstead & P. D. Cantino, subfam. nov

Type: *Cymaria* Bentham in Edwards’ Bot. Reg. 15: t. 1292. 1830.

Shrubs and subshrubs. Leaves simple, opposite, petiolate, elliptic or ovate to rhombic, crenate to crenulate or repand. Cymes axillary, lax, long-pedunculate, with secund, monochasial branches, sometimes grading into a terminal paniculiform thyrse. Calyx campanulate, accrescent, broadly campanulate to urceolate or subglobose in fruit, 5-lobed, lobes equal to subequal, triangular. Corolla white to yellowish, 2-lipped, posterior lip entire to deeply 2-lobed, anterior lip with median lobe largest. Stamens 4, didynamous (anterior pair longer), included or exserted, thecae divaricate, confluent at dehiscence; pollen tricolpate, tectate-perforate, suprareticulate, columellae simple to sparsely branched. Ovary shallowly 4-lobed; style sub-terminal, stigma lobes subequal to unequal; disc absent. Nutlets obovoid, reticulately ridged, pubescent, abscission-scar lateral, 0.4–0.6× the length of the nutlet.

This new subfamily consists of two small, tropical Asian genera: *Acrymia* (1 sp., Peninsular Malaysia) and *Cymaria* (2–3 spp., Hainan, Indo-China and Malesia)(species numbers and ranges from Harley *et al*.^16^). The two genera have been included in subfamily Ajugoideae^2^,^59^,^96^,^97^ or treated as *incertae sedis*^16^. A probable synapomorphy of Cymarioideae is its inflorescence structure: the cymes are axillary, lax, and long-pedunculate, with secund, monochasial branches^16^. This form of inflorescence is rare in the family but also occurs in *Garrettia*, where it apparently evolved independently.

When describing Cymarioideae, we realized that the type genus of the new subfamily, *Cymaria*, is currently without a type species. Bentham^98^ simultaneously named two species, *C. dichotoma* Benth. and *C. elongata* Benth., at the same time he described the genus, but he did not designate either as the type. *Cymaria* was recognized by several subsequent authors, and the two species were always listed in parallel without any type designation^1^,^2^,^97^,^99^. We take this opportunity to designate *C. dichotoma* as the type species for *Cymaria*, because it is more widely distributed and better represented in herbaria.

### Lamioideae Harley

The largely Old World subfamily Lamioideae is second in size only to Nepetoideae with over 60 genera and ca. 1200 species. Tribe Stachydeae is cosmopolitan in distribution, and Synandreae is endemic to North America. The other eight tribes are largely Eurasian, but four of them include some African species, and *Pogostemon* also occurs in Australia^16^,^21^,^23^. All Lamioideae have a gynobasic style, a synapomorphy that arose independently in Nepetoideae and Scutellarioideae^9^,^10^. Another possible synapomorphy is the presence of seed oils with an allenic component present^4^, but this character has been studied in too few species to be fully evaluated. Recent phylogenetic studies based on cpDNA sequence data have identified ten clades that have been ranked as tribes, with several genera unassigned to tribe^21^,^23^, but the monophyly of some of these tribes is not supported by nuclear DNA (PPR) data^26^. Further studies of relationships within some tribes (Synandreae—Scheen *et al*.^29^; Roy *et al*.^33^; Leucadeae—Scheen and Albert^100^; Phlomideae—Pan *et al*.^52^; Mathiesen *et al*.^101^; Salmaki *et al*.^30^; Lamieae—Bendiksby *et al*.^102^; Stachydeae—Salmaki *et al*.^31^; Gomphostemmateae—Xiang *et al*.^53^) have led to changes in the composition of some genera, acceptance of genera not recognized by Harley *et al*.^16^, and elimination of some genera that were recognized by Harley *et al*.^16^. In addition, Chen *et al*.^25^ showed that the formerly unplaced genus *Holocheila* belongs in Lamioideae. Besides *Holocheila*, four genera have been added to Lamioideae since 2004: *Rydingia* Scheen & V. A. Albert^103^, *Betonica* L.^21^, *Acanthoprasium* (Benth.) Spenn.^23^, and *Phlomoides* Moench^101^. Six genera recognized by Harley *et al*.^16^ are no longer recognized: *Alajja* Ikonn. (included in *Eriophyton* Benth.)^23^, *Sulaimania* Hedge & Rech. f. (included in *Moluccella* L.)^23^, *Pseudoeremostachys* Popov and *Lamiophlomis* Kudo (included in *Phlomoides*)^52^,^101^, *Bostrychanthera* Benth. (included in *Chelonopsis* Miq.)^102^, and *Stachyopsis* Popov & Vved. (included in *Eriophyton* Benth.)^104^. As a result of these changes, Lamioideae is now considered to have 62 genera (versus 63 recognized by Harley *et al*.^16^).

## Phylogenetic Nomenclature

Our analyses revealed five strongly supported but previously unnamed clades that contain subfamilies (labeled in Fig. 1). These clades warrant naming to facilitate communication about them, but there is no standard rank between family and subfamily. We therefore have given them unranked names, which are defined below, following the rules and recommendations of the draft PhyloCode^105^. For readers unfamiliar with phylogenetic nomenclature, the preface of the draft PhyloCode provides a good introduction (https://www.ohio.edu/phylocode/preface.html). The names of a variety of plant clades have been defined following the draft PhyloCode, including a set of major tracheophyte and angiosperm clades^106^, but there have been few previous applications of phylogenetic nomenclature to Lamiaceae. Cantino *et al*.^56^ provided phylogenetic definitions for some clade names within Lamiaceae to illustrate differences between phylogenetic and traditional nomenclature, but the PhyloCode did not yet exist, and the names and definitions in that paper were not intended to have any formal nomenclatural status. Salmaki *et al*.^31^ phylogenetically defined the name Eurystachys to accommodate the clade comprising the paraphyletic genus *Stachys* and ten other genera that nest within it. Phylogenetic definitions for the names Labiatae and Nepetoideae have been prepared by P. D. Cantino & R. G. Olmstead and will be published in *Phylonyms* (de Queiroz *et al*., in prep.).

### Cymalamiina B. Li, R. G. Olmstead & P. D. Cantino, new clade name

#### Definition

The smallest crown clade containing *Lamium purpureum* L. 1753 and *Cymaria dichotoma* Benth. 1930.

#### Primary reference phylogeny

Figure 2; see also Fig. 1 (this paper), Bendiksby *et al*.^23^ (Fig. 1), Chen *et al*.^25^ (Fig. 4), Roy and Lind qvist^26^ (Fig. 1a), and Chen *et al*.^43^ (Fig. 3, where *Cymaria* represents Cymarioideae).

#### Composition

Cymarioideae and Lamioideae.

#### Synapomorphies

Confluent anther thecae may be a synapomorphy. It is shared by Cymarioideae and one of the two basal subclades of Lamioideae (i.e., Pogostemoneae), but the anthers in the other basal subclade (i.e., the rest of Lamioideae) vary from distinct (e.g., Gomphostemmateae, Synandreae, *Galeopsis* L.) to confluent (e.g., *Colquhounia* Wall. and some Stachydeae). The closest outgroups to Cymalamiina (i.e., Scutellarioideae and Peronematoideae) have distinct thecae (except *Garrettia* in Peronematoideae), as do the more distant outgroups Premnoideae and *Tectona*; this character varies in Ajugoideae, another more distant outgroup. Given the distribution of the character states and its variability within many tribes of Lamioideae, it is not clear whether confluent thecae is a synapomorphy of Cymalamiina with a series of reversals within Lamioideae or, alternatively, that it evolved independently in Cymarioideae, Pogostemoneae, and in scattered other members of Lamioideae.

### Scutelamiina B. Li, R. G. Olmstead & P. D. Cantino, new clade name

#### Definition

The smallest crown clade containing *Lamium purpureum* L. 1753 and *Scutellaria galericulata* L. 1753.

#### Primary reference phylogeny

Figure 2; see also Fig. 1 (this paper), Bendiksby *et al*.^23^ (Fig. 1), Chen *et al*.^25^ (Fig. 4), and Chen *et al*.^43^ (Fig. 3).

#### Composition

Scutellarioideae, Cymarioideae and Lamioideae.

#### Synapomorphies

A four-lobed ovary appears to be a synapomorphy for Scutelamiina. It is shallowly four lobed in Cymarioideae and the more basal members of Scutellarioideae (though unlobed in *Holmskioldia*) and deeply lobed in Lamioideae and *Scutellaria*. A developmentally related feature, a schizocarpic fruit with four mericarps (“nutlets”), may be another synapomorphy of Scutelamiina. However, within its sister group (Peronematoideae), the 4-seeded capsule of *Garrettia* and *Peronema* breaks with pressure into four mericarps, possibly representing a stage in the evolution of the schizocarp of Scutelamiina. Another likely synapomorphy is suprareticulate (sometimes called bireticulate) pollen, which is found in all genera of Scutellarioideae and Cymarioideae and most genera of Lamioideae^90^,^107^,^108^,^109^. However the presence of suprareticulate pollen in *Garrettia* (Peronematoideae)^90^,^110^ and suprarugulose pollen in *Peronema*^110^ raises the possibility that this feature may be a synapomorphy for Perolamiina rather than Scutelamiina, though *Petraeovitex* and *Hymenopyramis* (the more distal genera within Peronematoideae) have psilate pollen^110^. Furthermore, suprareticulate sculpturing could be synapomorphic at an even more inclusive level because it occurs in some genera of Premnoideae (*Gmelina*, but not *Cornutia* or *Premna*, based on very few species^90^,^110^).

#### Comments

Cantino *et al*.^56^ applied the name Lamiina to this clade and provided a phylogenetic definition, but the names used in that paper were intended only to illustrate differences between phylogenetic and traditional rank-based nomenclature. They were not intended to have nomenclatural precedence under either system, and the PhyloCode did not yet exist.

### Perolamiina B. Li, R. G. Olmstead & P. D. Cantino, new clade name

#### Definition

The smallest crown clade containing *Lamium purpureum* L. 1753 and *Peronema canescens* Jack 1822.

#### Primary reference phylogeny

Figure 2; see also Fig. 1 (this paper), Bendiksby *et al*.^23^ (Fig. 1, where *Garrettia* represents Peronematoideae), Chen *et al*.^25^ (Fig. 4, where *Garrettia*, *Hymenopyramis* and *Petraeovitex* represent Peronematoideae), and Chen *et al*.^43^ (Fig. 3, where *Hymenopyramis*, *Petraeovitex* and *Peronema* represent Peronematoideae).

#### Composition

Peronematoideae, Scutellarioideae, Cymarioideae and Lamioideae.

#### Apomorphies

Supareticulate pollen may be a synapomorphy; see Scutelamiina.

### Viticisymphorina B. Li, R. G. Olmstead & P. D. Cantino, new clade name

#### Definition

The smallest crown clade containing *Vitex agnus-castus* L. 1753 and *Symphorema involucratum* Roxb. 1805 but not *Nepeta cataria* L. 1753, *Tectona grandis* L. f. 1782, *Premna serratifolia* L. 1771, *Ajuga reptans* L. 1753, and *Lamium purpureum* L. 1753.

#### Primary reference phylogeny

Figure 3; see also Fig. 1 (this paper), Bendiksby *et al*.^23^ (Fig. 1, where *Congea* represents Symphorematoideae, *Vitex* and *Petitia* represent Viticoideae), and Bramley *et al*.^47^ (Fig. 1, where *Sphenodesme* represent Symphorematoideae).

#### Composition

Viticoideae and Symphorematoideae.

#### Apomorphies

No non-molecular synapomorphies are known.

#### Comments

We intend this name to be applicable only if Symphorematoideae and Viticoideae are sister groups. Although our analyses strongly support the monophyly of this grouping, it is not supported in some other analyses^24^,^25^,^43^. For this reason, the definition includes external specifiers to make the name inapplicable under many alternative phylogenies.

### Calliprostantherina B. Li, R. G. Olmstead & P. D. Cantino, new clade name

#### Definition

The smallest crown clade containing *Callicarpa americana* L. 1753 and *Prostanthera lasianthos* Labillardiére 1806, but not *Vitex agnus-castus* L. 1753, *Nepeta cataria* L. 1753, *Tectona grandis* L. f. 1782, *Premna serratifolia* L. 1771, *Ajuga reptans* L. 1753, and *Lamium purpureum* L. 1753.

#### Primary reference phylogeny

Figure 3; see also Fig. 1 (this paper), Olmstead *et al*.^20^ (Fig. 1), Refulio-Rodriguez and Olmstead^15^ (Fig. 1A, where *Prostanthera* represents Prostantheroideae), and Chen *et al*.^43^ (Fig. 3, where *Prostanthera* and *Westringia* represent Prostantheroideae).

#### Composition

*Callicarpa* and Prostantheroideae.

#### Apomorphies

Branched trichomes and actinomorphic corollas, two characters that are infrequent in Lamiaceae, are shared by *Callicarpa* and Chloantheae (one of the two basal subclades of Prostantheroideae). Branched trichomes also occur in scattered species of the other subclade (Westringieae), increasing the likelihood that this feature characterizes Calliprostantherina. However, it could be apomorphic at a deeper level in the phylogeny since Symphorematoideae and *Tectona* also have branched hairs (see Fig. 1 for the relationship of the latter taxa to Calliprostantherina). Actinomorphic corollas also occur in *Tectona* and some genera of Symphorematoideae; furthermore, all species of Westringieae have zygomorphic flowers, weakening the hypothesis that actinomorphy is a synapomorphy for Calliprostantherina. There are similarities in pollen surface sculpturing between some species of *Callicarpa* and some genera of Prostantheroideae^111^, but there is considerable variation in both groups, and the polarity of the character is unknown.

#### Comments

Because the molecular support for this clade is only moderate and the potential morphological synapomorphies discussed above are not convincing, the definition is designed to become inapplicable under many alternative phylogenies (e.g., Bendiksby *et al*.^23^: Fig. 1).

**Type designation and new combinations**

***Cymaria*** **Bentham** in Edwards’s Bot. Reg. 15: t. 1292. 1830.*—***Type (here designated)**: *Cymaria dichotoma* Bentham in Edwards’ Bot. Reg. 15: t. 1292. 1830.

### *Vitex petitia* Bramley, nom. nov

Bas.: *Petitia domingensis* Jacq. in Enum. Syst. Pl.: 12. 1760.—**Lectotype (here designated):** Haiti, *Jacquin s.n.* (BM! fragment, barcode no. BM000992805).

**Note:**—A new name rather than a new combination has been created for *Petitia domingensis*, since the name *Vitex domingensis* Urb. & Ekman already exists (=*Pseudocarpidium domingense* (Urb. & Ekman) Moldenke). The epithet ‘petitia’ has been chosen to retain an obvious link to the original name of the species, which is quite common across the West Indies. The fragment of Jacquin’s specimen housed at the BM has been designated as lectotype, as suggested by annotation on the sheet by H. N. Moldenke according to d’Arcy^112^. Jacquin’s herbarium was reportedly bought by Banks and incorporated into his collections.

### *Vitex urbanii* (Ekman) Bramley, comb. nov

Bas.: *Petitia urbanii* Ekman in Ark. Bot. 21A(5): 94. 1927. —**Lectotype (here designated):** Haiti, Ile de la Tortue, steep limestone rocks west of Monillago Anglais, 22 May 1925, *E. L. Ekman H4096* (S! sheet number S04-2601; isolectotypes A!, B, F!, G!, K!, NY!, S!, UC!, US!).

**Note:**—One of the sheets at S [sheet number S04-2601] is designated as lectotype because E. L. Ekman was based there, and it has attached to it a handwritten note “it is a pleasure to dedicate this fine new species to Prof. Urban, the admirable botanist, the never tiring worker, and the best friend a man ever had”.

## Materials and Methods

### Choice of markers, taxon sampling and molecular data

Five chloroplast DNA markers—*matK*, *ndhF*, *rbcL*, *rps16*, and *trnL-F*—were employed in this study because (1) they have been widely used in phylogenetic reconstructions of Lamiaceae at generic, tribal or subfamilial level, and (2) many species of Lamiaceae have already been sequenced for these markers in previous molecular studies^9^,^10^,^21^,^22^,^23^,^24^,^25^,^26^,^27^,^28^,^29^,^30^,^31^,^32^,^33^,^34^,^35^,^36^,^37^,^38^,^39^,^40^,^41^,^42^,^43^,^44^,^45^,^46^,^47^,^48^,^49^,^50^,^51^,^52^,^53^,^101^. No comparable source of data exists for any nuclear DNA region for a broad sample of Lamiaceae.

The ingroup sample included representatives of all seven subfamilies and all ten genera *incertae sedis* recognized by Harley *et al*.^16^ and all 14 tribes recognized by Olmstead^18^. Nomenclature of Lamiaceae and Viticoideae s. str. followed Olmstead^18^ and Bramley *et al*.^47^, respectively. Initially, we downloaded data for all taxa of Lamiaceae with sequence information for any of the five gene regions deposited in Genbank as of August 2015. In the five subfamilies whose monophyly is well supported (viz., Ajugoideae, Lamioideae, Nepetoideae, Prostantheroideae and Scutellarioideae), sampling was designed to cover their genus-level diversity. Generally, genera with at least two sequenced regions were selected, and each selected genus was represented by one or two species. Particular emphasis was placed on sampling Symphorematoideae, Viticoideae s. str., all genera *incertae sedis*, and three genera formerly assigned to Viticoideae—*Cornutia*, *Gmelina*, and *Premna*. In three large genera—*Callicarpa*, *Premna*, and *Vitex*, sampling was designed to cover their morphological and geographic breadth. In total, 288 species representing 191 genera were included, representing approximately 78% of the genera of Lamiaceae. Five outgroup species were selected representing the closest relatives to Lamiaceae in Lamiales^12^,^13^,^14^,^15^. They are *Lindenbergia philippensis* (Cham. & Schltdl.) Benth. and *Pedicularis groenlandica* Retz. from Orobanchaceae, *Paulownia tomentosa* (Thunb.) Steud. from Paulowniaceae, *Mazus reptans* N. E. Br. from Mazaceae and *Phryma leptostachya* L. from Phrymaceae. Information on sampled taxa and Genbank accession numbers is assembled in Supplementary Table S1.

The five separate molecular data sets *matK*, *ndhF*, *rbcL*, *rps16* and *trnL-F* contained 202, 160, 170, 181, and 259 sequences with 54, 83, 59, 57, and 88 newly reported sequences, respectively. The dataset combining the five markers included 270 taxa (D270), with 39.65 % missing data. According to investigations by Wiens^113^ and Wiens and Moen^114^, the proportion of missing data should not affect the accuracy of the phylogenetic analysis; however, just to make sure, a reduced dataset was assembled including 155 taxa (D155) with at least three of the five regions or 50 % of the total aligned sequence length available for each terminal taxon. The total amount of missing data in D155 was 23.51 %. For most species in the combined datasets, data were available for all five regions, but there were some genera of Ajugoideae, Lamioideae, Nepetoideae, Prostantheroideae, and Scutellarioideae in which different species were used for different gene regions. When data were pooled in this way, generic names, rather than species names, were used to represent the combined sequences in the phylogenetic trees.

### DNA extraction, amplification, and sequencing

The 2x CTAB method of Doyle and Doyle^115^ was used to extract total genomic DNA of the samples with silica dried leaf tissue, and DNEasy^®^ Plant Mini Kit (QIAGEN^®^, Valencia, California, USA) was used for herbarium materials according to the manufacturer’s specifications. The DNA extracts were dissolved in TE buffer and preserved at −20 °C for further use.

Primer pairs used in Polymerase chain reaction (PCR) amplification of the five regions are listed in Table S2 with their sequences and references. The PCR reaction system and amplification protocol were identical for all five fragments. PCR reactions used 2.5 μL sample DNA, 0.5 μL Dream *Taq* DNA polymerase, 5 μL 10 × DreamTaq Green Buffer, 5 μL dNTP Mix (2 mM each), 1 μL of bovine serum albumin (BSA, 20 mg/mL), 1 μL of each primer in a final reaction volume of 50 μL. The PCR program was as follows: an initial template denaturation at 94 °C for 5 min, 35 cycles of 30 second denaturation at 94 °C, 1 minute primer annealing at 52 °C, 1.5 min extension at 72 °C, with a final extension of 8 min at 72 °C. Sequencing was done by the Invitrogen sequencing service (Invitrogen, commercial sequencing facility, Guangzhou, China) using the same primers for PCR amplifications.

### Sequence alignment and phylogenetic analyses

Sequencher v.4.5^116^ was used to evaluate chromatograms for base confirmation and to edit contiguous sequences. All DNA sequences were initially aligned using Clustal X v.2.0.^117^ and adjusted manually in BioEdit Sequence Alignment Editor v.7.0.0^118^.

The separate data sets were first analyzed using Maximum parsimony (MP) and Maximum likelihood (ML) methods, with gaps treated as simple indels determined by the program Gapcoder^119^ and added to the matrix as binary presence/absence characters. The combined data sets D270 and D155 were analyzed using MP, ML and Bayesian inference (BI) methods with gaps treated either as missing data or as simple indels.

MP analyses were conducted using PAUP* v.4.0b10^120^ with all characters unordered and equally weighted. Heuristic search was conducted using 1000 random addition sequence replicates, tree-bisection-reconnection (TBR) branch swapping, MulTrees in effect, and steepest descent off. Bootstrap support values (BS) were estimated using a heuristic search strategy with 500 bootstrap replicates and 1000 random sequences additions.

ML analyses were performed on the web server RAxML Black Box^121^. Before each submission, the “Maximum likelihood search” and “Estimate proportion ofinvariable sites” options were selected, with a total of 1000 bootstrap replicates performed.

BI analysis was executed using MrBayes version 3.2.2^122^ on the CIPRES Science Gateway^123^ with the default parameters. The best substitution types (Nst) and rate distribution models (rates) were determined by the Akaike information criterion (AIC) using Model Test v.3.7^124^ with the hierarchical likelihood ratio tests. Four Markov chain Monte Carlo (MCMC) chains were run, each beginning with a random tree and sampling one tree every 1000 generations for 30 000 000 generations. Mixing, convergence and a suitable burn-in were assessed with the statistics provided by the program and with Tracer v. 1.6^125^. Post burn-in samples from the four runs were merged using LogCombiner v1.7.5. (available at http://beast.bio.ed.ac.uk/, LogCombiner) prior to the calculation of a 50 % majority-rule consensus tree.

## Additional Information

**How to cite this article**: Li, B. *et al*. A large-scale chloroplast phylogeny of the Lamiaceae sheds new light on its subfamilial classification. *Sci. Rep.* **6**, 34343; doi: 10.1038/srep34343 (2016).

## Supplementary Material

Supplementary Information

## Figures and Tables

**Table 1:** Properties of data partitions used in this study and tree statistics.

Data matrix	Number of sequences	New reported sequences	Number of aligned positions	Number of informative substitutions	Number of indels	Number of informative indels	Proportion of missing data	Tree length	Consistency index (CI)	Retention index (RI)	Model
*matK*	202	54	1578	613	19	10	25.54%	2384	0.53	0.87	TVM + I + G
*ndhF*	160	83	2108	765	23	9	19.13%	3891	0.43	0.75	GTR + I + G
*rbcL*	170	59	1400	251	2	0	11.57%	1282	0.34	0.75	TVM + I + G
*rps16*	181	57	926	375	89	56	1.62%	1602	0.55	0.88	GTR + G
*trnL-F*	259	88	918	398	123	64	1.67%	1801	0.52	0.88	GTR + G
D270	270		6930	2402	256	139	39.65%	11084	0.47	0.83	GTR + I + G
D155	155		6930	2168	218	123	23.51%	9381	0.51	0.80	GTR + I + G

**Table 2:** Comparison of support values for subfamilial or above nodes in the different analyses.

Clade	matK	ndhF	rbcL	rps16	trnL-F	D270		D155	
						gaps treated as simple indels	gaps treated as missing data	gaps treated as simple indels	gaps treated as missing data
Ajugoideae	100, 98	100, 100	85, 61	100, 98	98, 88	1.00, 100, 100	1.00, 100, 100	1.00, 100, 100	1.00, 100, 100
*Callicarpa*	99, 94	100, 100	100, 92	100, 92	99, 79	1.00, 100, 100	1.00, 100, 100	100, 100, 1.00	1.00, 100, 100
Calliprostantherina	−, −	95, 62	−, −	−, −	−, −	1.00, 91, 60	1.00, 88, 59	0.96, 75, −	0.93, 67, −
Cymalamiina	100, 97	100, 99	90, 81	100, 98	98, 87	1.00,100, 99	1.00,100, 99	100, 100, 1.00	100, 100, 1.00
Cymarioideae	−, −	95, 74	−, −	56, 51	89, 75	1.00, 93, 83	1.00, 89, 79	1.00, 97, 86	0.98, 87, 80
Lamioideae	−, −	100, 95	−, −	95, 90	79, 74	100, 100, 1.00	1.00, 100, 99	100, 100, 1.00	1.00, 100, 99
Nepetoideae	100, 100	100, 100	100, 100	100, 100	100, 100	100, 100, 1.00	1.00, 100, 100	100, 100, 1.00	100, 100, 1.00
Perolamiina	−, −	100, 91	−, −	100, 88	76, 56	1.00, 100, 92	1.00, 96, 89	1.00, 100, 90	1.00, 94, 76
Peronematoideae	100, 93	93, 85	100, 99	79, 61	99, 86	100, 100, 1.00	1.00, 100, 99	100, 100, 1.00	1.00, 100, 99
Premnoideae	−, −	92, 91	−, −	−, −	−, −	1.00, 100, 97	1.00, 100, 96	1.00, 97, 91	1.00, 95, 90
Prostantheroideae	100, 100	100, 100	100, 100	100, 100	100, 98	100, 100, 1.00	1.00, 100, 100	100, 100, 1.00	1.00, 100, 100
Scutelamiina	−, −	100, 93	52, −	98, 85	100, 94	100, 100, 100	1.00, 100, 100	100, 100, 1.00	100, 100, 1.00
Scutellarioideae	100, 100	100, 100	88, 76	100, 100	100, 99	100, 100, 1.00	1.00, 100, 100	100, 100, 1.00	1.00, 93, 69
Symphoremoideae	100, 100	100, 100	100, 98	100, 100	100, 100	100, 100, 1.00	1.00, 100, 100	100, 100, 1.00	100, 100, 1.00
*Tectona*	100, 100	100, 100	100, 99	100, 100	100, 100	100, 100, 1.00	1.00, 100, 100	100, 100, 1.00	100, 100, 1.00
Viticoideae	100, 99	100, 100	92, 85	100, 100	100, 100	100, 100, 1.00	1.00, 100, 100	100, 100, 1.00	100, 100, 1.00
Viticisymphorina	81, 66	−, −	86, 67	98, 78	100, 96	100, 100, 1.00	1.00, 100, 100	100, 100, 1.00	1.00, 100, 99

The numbers in *matK*, *ndhF*, *rbcL*, *rps16* and *trnL-F* were bootstrap support values in ML and MP analysis respectively, with gaps treated as simple indels. The numbers in D270 and D155 were posterior probabilities values in BI analysis, and bootstrap support values in ML and MP analysis, respectively. “−” Indicates support values of less than 50% in MP or ML analysis, and posterior probabilities value less than 0.90 in BI analysis.
