# Supplementary material for: A large-scale chloroplast phylogeny of the Lamiaceae sheds new light on its subfamilial classification
Source: Sci Rep. 2016 Oct 17;6:34343. doi: 10.1038/srep34343 (PMC5066227; doi:10.1038/srep34343)

A large-scale chloroplast phylogeny of the Lamiaceae sheds new light on its subfamilial classification

Bo Li[1,+], Philip D. Cantino[2,+], Richard G. Olmstead[3,+], Gemma L. C. Bramley[4], Chun-Lei Xiang[5], Zhong-Hui Ma[6], Yun-Hong Tan[7] & Dian-Xiang Zhang[8,*]

[1] College of Agronomy, Jiangxi Agricultural University, Nanchang, 330045, Jiangxi, P. R. China

[2] Department of Environmental and Plant Biology, Ohio University, Athens, Ohio 45701-2979, U. S. A.

[3] Department of Biology and Burke Museum, University of Washington, Box 355325, Seattle, Washington 98195-5325, U. S. A.

[4] Herbarium, Royal Botanic Gardens Kew, Richmond, Surrey, TW9 3AE, U.K.

[5] Key Laboratory for Plant Diversity and Biogeography of East Asia, Kunming Institute of Botany, Chinese Academy of Sciences, Kunming 650201, Yunnan, P. R. China

[6] College of Agriculture, Guangxi University, Nanning 530004, Guangxi, P. R. China

[7] Key Laboratory of Tropical Forest Ecology, Xishuangbanna Tropical Botanical Garden, Chinese Academy of Sciences, Mengla 666303, Yunnan, P. R. China

[8] South China Botanical Garden, Chinese Academy of Sciences, Guangzhou 510650, Guangdong, P. R. China

[+]these authors contributed equally to this work

[*] Corresponding author: dx-zhang@scbg.ac.cn

keywords: cpDNA, new subfamily, phylogenetic nomenclature, phylogenetic systematics, taxonomy

Supplementary Table S1. List of taxa included in the phylogeny of Lamiaceae study. "Taxa" refers spceies or genus included in the combined dataset D270; a single generic name indicates that different gene regions were represented by different species of the genus; taxa marked by symbol "*" means that the taxa was excluded in the reduced dataset D155. "Classification" indicates the families and subfamilies to which the ingroup and outgroup species belongs; LA represents Lamiaceae; Aj, Cy, La, Ne, Per, Pre, Pro, Sc, Sy and Vi connected with LA by "–" represent the ten subfamilies of Lamiaceae recognized by Harley *et al.*[1], Olmstead[2] and the present study, viz., Ajugoideae, Cymarioideae, Lamioideae, Nepetoideae, Peronematoideae, Premnoideae, Prostantheroideae, Scutellarioideae, Symphorematoideae and Viticoideae, respectively. "matK" "ndhF" "rbcL" "rps16" and "trnL-F" present GenBank accession numbers for each molecular marker; sequences generated in this study are marked by symbol "●"; "–" refers to missing sequence.

Taxa	**Classification**	**matK**	**ndhF**	**rbcL**	**rps16**	**trnL-F**
**Acanthomintha lanceolata* Curran	LA-Ne	GU381737	-	-	-	DQ667522
**Achyrospermum fruticosum* Benth.	LA-La	HQ911420	-	-	FJ854004	FJ854251, FJ854138
**Acrotome inflata* Benth.	LA-La	HQ911533	-	-	EU138225	EU138378, EU138300
Acrymia ajugiflora Prain	LA-Cy	-	●	-	HQ911584	●
Aegiphila Jacq.	LA-Aj	JQ588060 *A. panamensis* Moldenke	AY310121 *A. costaricensis* Moldenke	JQ594359 *A. costaricensis* Moldenke	JQ322783 *A. sellowiana* Cham.	FJ952070 *A. alba* Moldenke
**Aeollanthus densiflorus* Ryding	LA-Ne	-	-	-	AJ505328	AJ505435
**Agastache foeniculum* (Pursh) Kuntze	LA-Ne	AY840146	-	Z37381	-	JQ669022
Ajuga decumbens Thunb.	LA-Aj	AF315299	JQ322521	JQ322527	-	EF153683
Ajuga reptans L.	LA-Aj	HQ911379	L36391	Z37385	HQ911574	HQ911643, HQ911712
**Ajugoides humilis* (Miq.) Makino	LA-La	HQ9114525	-	-	HQ911609	HQ911678, HQ911746
**Alvesia rosmarinifolia* Welw.	LA-Ne	-	-	-	AJ505329	AJ505436
**Amasonia sp.*	LA-Aj	-	AY310122	-	-	FJ952066
**Amethystea caerulea* L.	LA-Aj	-	AY115882	-	-	●
**Anisochilus pallidus* Wall.	LA-Ne	-	-	JQ933218	AJ505331	AJ505438
Anisomeles indica (L.) Kuntze	LA-La	HQ911395	●	FJ513149	FJ854012	FJ854259, FJ854146

Ballota africana (L.) Benth.	LA-La	FQ911493	-	AM234997	FJ854014	FJ854261, FJ854148
Ballota nigra L.	LA-La	HQ911497	-	HM849806	FJ854017	HQ911698, FJ854151
**Basilicum polystachyon* (L.) Moench	LA-Ne	-	-	-	AJ505332	AJ505439
**Blephilia ciliata* (L.) Raf. ex Benth.	LA-Ne	GU381743	-	-	-	GU381580
**Bostrychanthera deflexa* Benth.	LA-La	HQ911423	-	-	FJ854020	FJ854267, FJ854154
**Brachysola coerulea* (F. Muell. & Tate) Rye	LA-Pro	-	●	-	-	●
**Brazoria arenaria* Lundell	LA-La	HQ911431	-	-	FJ854021	EF546967, EF546891
Callicarpa americana L.	LA	●	●	●	●	●
Callicarpa angusta Schauer	LA	●	●	●	●	●
Callicarpa arborea Roxb.	LA	●	●	●	●	●
Callicarpa bodinieri H. Lév.	LA	●	●	●	●	●
Callicarpa brevipes (Benth.) Hance	LA	●	●	●	●	●
Callicarpa cathayana C. H. Chang	LA	●	●	●	●	●
Callicarpa dichotoma (Lour.) K. Koch	LA	●	●	●	●	●
Callicarpa erioclona Schauer	LA	●	●	●	●	●
Callicarpa glandulosa H. R. Fletcher	LA	●	●	●	●	●
Callicarpa hispida (Moldenke) Bramley	LA	●	●	●	●	●
Callicarpa japonica Thunb.	LA	●	●	●	●	●
Callicarpa kwangtungensis Chun	LA	●	●	●	●	●
Callicarpa maingayi King & Gamble	LA	●	●	●	●	●
Callicarpa mollis Siebold & Zucc.	LA	HQ384498	AY310134	HQ384868	HQ385145	HQ412928
Callicarpa pedunculata R. Br.	LA	●	●	●	●	JQ669025
Callicarpa peii C. H. Chang	LA	●	●	●	●	●
Callicarpa pentandra Roxb.	LA	●	●	●	●	●
Callicarpa scandens (Moldenke) Govaerts	LA	-	●	●	●	●
**Capitanopsis angustifolia* (Moldenke) Capuron	LA-Ne	-	-	-	AJ505333	AJ505440
Caryopteris incana (Thunb. ex	LA-Aj	AF315295	U78681	U28869	-	JF3013596

Houtt.) Miq.					
**Catoferia chiapensis* A. Gray ex Benth.	LA-Ne	-	-	-	AJ505414	AJ505537
**Cedronella canariensis* (L.) Webb & Berthel.	LA-Ne	HM850788	-	HM849871	-	AY506622
**Chaiturus marrubiastrum* (L.) Ehrh. ex Rchb.	LA-La	HQ911464	-	-	FJ854022	FJ854268, FJ854155
**Chamaesphacos ilicifolius* Schrenk	LA-La	HQ911549	-	-	FJ854023	FJ854269, FJ854156
**Chelonopsis moschata* Miq.	LA-La	HQ911425	-	-	FJ854025	FJ854270, FJ854157
**Chloanthes parviflora* Walp.	LA-Pro	-	●	-	-	●
Clerodendrum japonicum (Thunb.) Sweet	LA-Aj	AF315297	AY115886	GQ436521	HQ911575	HQ911644, HQ911713
Clerodendrum trichotomum Thunb.	LA-Aj	AF477760	AF130146	HQ384865	HQ385140	FJ952051
Clinopodium vulgare L.	LA-Ne	AY840153	-	HM849904	AJ505426	DQ667513
Colebrookea oppositifolia Sm.	LA-La	HQ911391	U78688	U78712	HQ911587	HQ911657, HQ911725
**Coleus* Lour.	LA-Ne	-	-	HQ839694<br>*C. carnosifolius* (Hemsl.) Dunn	FJ593291<br>*C. xanthanthus* C. Y. Wu & Y. C. Huang	FJ593411<br>*C. xanthanthus* C. Y. Wu & Y. C. Huang
**Collinsonia canadensis* L.	LA-Ne	AY840148	-	Z37387	-	AY570453
Comanthosphace japonica (Miq.) S. Moore	LA-La	HQ911409	●	-	FJ854031	FJ854274, FJ854161
Congea tomentosa Roxb. 1	LA-Sy	HQ384499	HQ384815	HQ384869	AJ505411	HQ412929
Congea tomentosa Roxb. 2	LA-Sy	-	U78689	U28870	-	AJ505530
**Conradina canescens* A. Gray	LA-Ne	GU381749	-	-	-	DQ667438
Cornutia pyramidata L. 1	LA-Pre	HQ911375	●	JQ592284	HQ911571	●
Cornutia pyramidata L. 2	LA-Pre	●	●	●	●	●
Craniotome furcata (Link) Kuntze	LA-La	HQ911392	-	JQ933281	FJ854032	FJ854275, FJ854162
**Cuminia eriantha* (Benth.) Benth.	LA-Ne	GU381729	-	-	-	GU381566
**Cunila origanoides* (L.) Britton	LA-Ne	GU381752	-	-	-	JQ669034
**Cyanostegia angustifolia* Turcz.	LA-Pro	-	●	-	-	●
**Cyclotrichium niveum* (Boiss.) Manden. & Scheng.	LA-Ne	GU381691	-	-	-	GU381525
Cymaria acuminata Decne.	LA-Cy	KF509859	KF509863	KF509870	FJ853997	FJ854244 FJ854131
Cymaria dichotoma Benth.	LA-Cy	HQ911388	●	-	FJ853998	FJ854245 FJ854132
**Dauphinea brevilabra* Hedge	LA-Ne	-	-	-	AJ505334	AJ505441

**Dicrastylis fulva* Drumm. ex Harv.	LA-Pro	-	●	-	-	●
**Dorystaechas hastata* Boiss. & Heldr. ex Benth.	LA-Ne	-	-	AY570383	-	AY570454
**Dracocephalum moldavica* L.	LA-Ne	-	-	Z37389	-	AY506625
Elsholtzia stauntonii Benth.	LA-Ne	-	U786909	U28872	AJ505406	AJ505526
Eremostachys Bunge	LA-La	HQ911485<br>*E. molucelloides* Bunge	-	AF501985<br>*E. labiosa* Bunge	GU993165<br>*E. laevigata* Bunge	GU993260, GU993069<br>*E. laevigata* Bunge
Eriophyton wallichii Benth.	LA-La	HQ911462	-	JF941487	FJ854034	FJ854277, FJ854164
**Eurysolen gracilis* Prain	LA-La	HQ911402	-	-	HQ911593	HQ911665, HQ911733
Faradaya F. Muell.	LA-Aj	HQ911381<br>*F. amicorum* (Seem.) Seem.	AY310127<br>*F. splendida* F. Muell.	JF738822<br>*F. splendida* F. Muell.	HQ911576<br>*F. amicorum* (Seem.) Seem.	HQ911645, HQ911714<br>*F. amicorum* (Seem.) Seem.
**Fuerstia africana* T. C. E. Fr.	LA-Ne	-	-	-	AJ505427	AJ505550
Garrettia siamensis H. R. Fletcher 1	LA-Per	●	●	●	●	●
Garrettia siamensis H. R. Fletcher 2	LA-Per	●	●	●	●	●
Garrettia siamensis H. R. Fletcher 3	LA-Per	●	●	●	●	●
* *Garrettia siamensis* H. R. Fletcher 4	LA-Per	-	-	-	HQ911579	HQ911648 HQ911717
Glechoma hederacea L.	LA-Ne	AY840143	U78691	Z37391	-	AY570455
Gmelina arborea Roxb. ex Sm. 1	LA-Pre	●	●	●	●	●
Gmelina arborea Roxb. ex Sm. 2	LA-Pre	●	●	●	●	●
Gmelina hainanensis Oliv.	LA-Pre	●	JQ322518	JQ322524	●	●
Gmelina hystrix Schult. ex Kurz	LA-Pre	-	U78692	U28873	AJ505407	AJ505527
Gmelina philippensis Cham.	LA-Pre	●	●	●	●	●
**Gomphostemma javanicum* (Blume) Benth.	LA-La	-	-	AF501986	FJ854038	AF502028, EF546860
**Hanceola sinensis* (Hemsl.) Kudô	LA-Ne	-	-	-	FJ593294	FJ593415
Haplostachys haplostachya (A. Gray) H. St. John	LA-La	HQ911565	-	AF501987	FJ854039	KF235643, KF235696
**Haumaniastrum katangense* (S. Moore) P. A. Duvign. & Plancke	LA-Ne	-	-	-	AJ505417	AJ505540
**Hedeoma costata* Hemsl.	LA-Ne	GU381755	-	-	-	DQ667436
**Hemiandra pungens* R.Br.	LA-Pro	-	●	-	-	●
Hemigenia R.Br.	LA-Pro	HQ911374<br>*H. pedunculata* Diels	●<br>*H. incana* (Lindl.) Benth.	-	HQ911570<br>*H. pedunculata* Diels	●<br>*H. incana* (Lindl.) Benth.

**Hemiphora elderi* (F. Muell.) F.Muell.	LA-Pro	-	●	-	-	●
**Hesperozygis nitida* (Benth.) Epling	LA-Ne	GU381730	-	-	-	GU381567
**Hoehnea epilobioides* (Epling) Epling	LA-Ne	GU381731	-	-	-	DQ667497
Holmskioldia sanguinea Retz.	LA-Sc	HQ911382	U78693	U28874	HQ911581	HQ911651, HQ911719
Holocheila longipedunculata S. Chow	LA-La	AF315304	KF509860	KF509867	KF509871	KF509874, KF509880
**Holostylon katangense* Robyns & Lebrun	LA-Ne	-	-	-	AJ505335	AJ505443
**Horminum pyrenaicum* L.	LA-Ne	AY840177	-	-	-	AY570456
**Hoslundia opposita* Vahl	LA-Ne	-	-	-	AJ505428	AJ505551
**Huxleya linifolia* Ewart & B. Rees	LA-Aj	-	AY310128	-	-	-
Hymenopyramis brachiata Wall. ex Griff.	LA-Per	●	●	●	●	●
**Hymenopyramis cana* Craib	LA-Per	-	KF509865	-	-	KF509879 KF509885
Hymenopyramis siamensis Craib	LA-Per	●	●	●	●	●
**Hypenia macrantha* (A. St.-Hil. ex Benth.) Harley	LA-Ne	-	-	-	AJ505336	AJ505445
**Hypogomphia turkestana* Bunge	LA-La	HQ911551	-	-	HQ911634	HQ911703, HQ911774
Hyptis Jacq.	LA-Ne	JF357886<br>*H. brevipes* Poit.	-	JQ592289<br>*H. brevipes* Poit.	AJ505337<br>*H. capitata* Jacq.	AJ505448<br>*H. brevipes* Poit.
Hyssopus L.	LA-Ne	-	●<br>*H. seravschanicus* (Dubj.) Pazij	Z37395<br>*H. officinalis* L.	-	●<br>*H. seravschanicus* (Dubj.) Pazij
Isodon (Schrad. ex Benth.) Spach	LA-Ne	JF954195<br>*I. bulleyanus* (Diels) Kudô	-	HQ839688<br>*I. rubescens* (Hemsl.) H. Hara	JQ389539<br>*I. rubescens* (Hemsl.) H. Hara	JQ389559<br>*I. rubescens* (Hemsl.) H. Hara
**Isoleucas arabica* O. Schwartz	LA-La	HQ911508	-	-	EU138227	EU138380, EU138303
**Kalaharia uncinata* (Schinz) Moldenke	LA-Aj	-	AY310130	-	-	FJ952076
Karomia speciosa (Hutch. & Corbishley) R. Fern.	LA-Aj	JF270836	AY310131	JF265489	-	●
**Kurzamra pulchella* (Clos) Kuntze	LA-Ne	GU381758	-	-	-	GU381598
**Lachnostachys eriobotrya* (F. Muell.) Druce	LA-Pro	-	●	-	-	●
**Lagochilus hirtus* Fisch. & C. A. Mey.	LA-La	HQ911469	-	-	FJ854041	FJ854280, FJ854168

Lagopsis (Bunge ex Benth.) Bunge	LA-La	HQ911472<br>*L. marrubiastrum* (Stephan) Ikonn.-Gal.	-	HQ839687<br>*L. supina* (Stephan ex Willd.) Ikonn.-Gal.	HQ911618<br>*L. supina* (Stephan ex Willd.) Ikonn.-Gal.	HQ911689, HQ911760<br>*L. supina* (Stephan ex Willd.) Ikonn.-Gal.
Lamium purpureum L.	LA-La	JF7798771	U78694	Z3740320	HQ385141	JF78002776
Lavandula L.	LA-Ne	GU391427<br>*L. angustifolia* Mill.	●<br>*L. lanata* Boiss.	Z37404<br>*L. angustifolia* Mill.	JQ322781<br>*L. stoechas* L.	AY570457<br>*L. angustifolia* Mill.
Leonotis leonurus (L.) R. Br.	LA-La	HQ911521	-	AM234998	JX0732795	EU138382, EU138305
Leonurus sibiricus L.	LA-La	HQ911471	-	HQ839683	FJ854045	EF546930, EF546852
**Lepechinia calycina* (Benth.) Epling	LA-Ne	-	-	AY570386	-	JF301375
Leucas R. Br.	LA-La	HQ911520<br>*L. minimifolia* Chiov.	-	AM234999<br>*L. capensis* (Benth.) Engl.	EU138266<br>*L. minimifolia* Chiov.	EU138419, EU138342<br>*L. minimifolia* Chiov.
Lindenbergia philippensis (Cham. & Schltdl.) Benth.	Orobanchaceae	AF051990	AF123686	AF123664	AJ609169	AJ608586
**Lycopus europaeus* L.	LA-Ne	AY840154	-	HM850150	-	GU381478
**Macbridea alba* Chapm.	LA-La	HQ911428	-	-	HQ911598	EF546962, EF546885
Marrubium vulgare L.	LA-La	HQ911499	U78695	U28875	EU138294	EU138443, EU138366
**Marsypianthes chamaedrys* (Vahl) Kuntze	LA-Ne	JQ588075	-	JQ592298	-	JF357815
**Matsumurella tuberifera* (Makino) Makino	LA-La	HQ911453	-	-	HQ911610	HQ911679, HQ911747
Mazus reptans N. E. Br.	Mazaceae	HQ384502	HQ384817	HQ384872	HQ385147	AF479004
Melissa officinalis L.	LA-Ne	HM850795	●	Z37413	AJ505410	DQ667477
Melittis melissophyllum L.	LA-La	HQ911534	-	HE963562	HQ911633	HQ911702, HQ911773
Mentha L.	LA-Ne	GU381686<br>*M.* × *rotundifolia* (L.) Huds.	U78696<br>*M.* × *rotundifolia* (L.) Huds.	Z37417<br>*M.* × *rotundifolia* (L.) Huds.	FJ593336<br>*M. sp.*	GU381520<br>*M.* × *rotundifolia* (L.) Huds.
**Micromeria juliana* (L.) Benth. ex Rchb.	LA-Ne	AY840159	-	JQ933407	-	JQ669056
Microcorys R.Br.	LA-Pro	HQ911372<br>*M. loganiacea* F. Muell.	●<br>*M. exserta* Benth.	-	HQ911568<br>*M. loganiacea* F. Muell.	●<br>*M. exserta* Benth.
**Microtoena* Prain	LA-La	HQ911393<br>*M. patchoulii* (C. B. Clarke ex Hook. f.) C. Y. Wu & S. J. Hsuan	-	-	FJ854052<br>*M. patchoulii* (C. B. Clarke ex Hook. f.) C. Y. Wu & S. J. Hsuan	HQ911658, HQ911726<br>*M. delavayi* Prain
**Minthostachys mollis* (Benth.) Griseb.	LA-Ne	GU381763	-	-	-	JQ669059
**Moluccella* L.	LA-La	HQ911491<br>*M. spinosa* L.	-	-	FJ854055<br>*M. laevis* L.	EU138444, EU138367<br>*M. laevis* L.

**Monarda fistulosa* L.	LA-Ne	GU381745	-	Z37419	-	DQ667506
**Monardella odoratissima* Benth.	LA-Ne	GU381744	-	-	-	GU381581
**Mosla chinensis* Maxim.	LA-Ne	HQ839706	-	FJ513158	-	-
Nepeta L.	LA-Ne	HQ593369 *N. cataria* L.	-	Z37421 *N. cataria* L.	AJ505326 *N. straussii* Hausskn. & Bornm.	DQ667487 *N. cataria* L.
**Newcastelia cladotricha* F. Muell.	LA-Pro	-	●	-	-	●
**Obtegomeria caerulescens* (Benth.) Doroszenko & P. D. Cantino	LA-Ne	GU381759	-	-	-	GU381599
Ocimum L.	LA-Ne	AY177670 *O. basilicum* L.	JN686623 *O. americanum* L.	Z37425 *O. basilicum* L.	FJ593338 *O. basilicum* L.	AY570462 *O. basilicum* L.
Ombrocharis dulcis Hand.-Mazz.	LA-Ne	-	●	●	-	●
Oncinocalyx betchei F. Muell.	LA-Aj	-	U78685	U31458	-	JN408590
Origanum vulgare L.	LA-Ne	GU381802	JX880022	Z37427	AJ505422	AY570463
Orthosiphon aristatus (Blume) Miq.	LA-Ne	JN119569	-	KF496653	FJ593340	FJ593460
Otostegia tomentosa A. Rich.	LA-La	HQ911515	-	AF501988	EU138286	EU138440, EU138364
**Oxera pulchella* Labill.	LA-Aj	-	AY310132	-	-	FJ952077
**Panzerina lanata* (L.) Soják	LA-La	HQ911474	-	-	FJ854058	FJ854289, FJ854176
**Paraphlomis javanica* (Blume) Prain	LA-La	HQ911451	-	-	FJ854060	FJ854292, FJ854178
Paulownia tomentosa (Thunb.) Steud.	Paulowniaceae	AJ429339	L36406	L36447	AJ431051	AY423122
Pedicularis groenlandica	Orobanchaceae	HQ384503	HQ384818	HQ384873	HQ385148	HQ412930
**Pentapleura subulifera* Hand.-Mazz.	LA-Ne	GU381781	-	-	-	GU381625
Perilla frutescens (L.) Britton	LA-Ne	AF315307	JN686622	FJ513160	JN686530	DQ667439
Peronema canescens Jack	LA-Per	-	●	●	●	●
**Perovskia abrotanoides* Kar.	LA-Ne	-	-	Z37429	-	AY506615
Petitia domingensis Jacq.	LA-Vi	HQ911376	U78697	U28878	HQ911572	●
Petraeovitex multiflora (Sm.) Merr. 1	LA-Per	●	●	●	●	●
**Petraeovitex multiflora* (Sm.) Merr. 2	LA-Per	-	KF509864	-	-	KF509878 KF509884
Petraeovitex sp.	LA-Per	●	●	●	●	●

**Phlomidoschema parviflorum* (Benth.) Vved.	LA-La	HQ911546	-	-	FJ854062	FJ854293, FJ854179
Phlomis fruticosa L.	LA-La	HQ911475	-	HM850253	GU993105	GU993201, GU993010
Phlomoides Moench	LA-La	HQ911479 *P. rotata* (Benth. ex Hook. f.) Mathiesen	-	JQ933445 *P. melanantha* (Diels) Kamelin & Makhm.	GU993145 *P. rotata* (Benth. ex Hook. f.) Mathiesen	GU993241, GU993050 *P. rotata* (Benth. ex Hook. f.) Mathiesen
Phryma leptostachya L.	Phrymaceae	AJ429341	AJ617586	U28881	DQ532450	DQ532486
Phyllostegia Benth.	LA-La	HQ911566 *P. velutina* (Sherff) H. St. John	-	AF501991 *P. macrophylla* (Gaudich.) Benth.	HQ911635 *P. velutina* (Sherff) H. St. John	HQ911704, HQ911775 *P. velutina* (Sherff) H. St. John
**Physopsis spicata* Turcz.	LA-Pro	-	●	-	-	●
Physostegia virginiana (L.) Benth.	LA-La	HQ911436	L364079	L14405	HQ911602	HQ911671, HQ911738
**Piloblephis rigida* (W. Bartram ex Benth.) Raf.	LA-Ne	GU381746	-	-	-	GU381584
**Pityrodia loxocarpa* (F. Muell.) Druce	LA-Pro	-	●	-	-	●
**Platostoma kerrii* Suddee & A. J. Paton	LA-Ne	-	-	-	AJ505370	AJ505491
Plectranthus L'Hér.	LA-Ne	AY840166 *P. fruticosus* L'Hér.	U78698 *P. barbatus* Andrews	U28882 *P. barbatus* Andrews	AJ505378 *P. barbatus* Andrews	AJ505500 *P. barbatus* Andrews
**Pogogyne zizyphoroides* Benth.	LA-Ne	GU381735	-	-	-	GU381572
Pogostemon Desf.	LA-La	EF529565 *P. cablin* (Blanco) Benth.	U78699 *P. cablin* (Blanco) Benth.	L14406 *P. cablin* (Blanco) Benth.	HQ911592 *P. benghalensis* (Burm. f.) Kuntze	HQ911663, HQ911731 *P. benghalensis* (Burm. f.) Kuntze
**Poliomintha incana* (Torr.) A. Gray	LA-Ne	GU381741	-	-	-	GU381578
Prasium majus L.	LA-La	HQ911541	U78700	U31459	FJ854072	FJ854300, FJ854187
Premna aff. serratifolia L.	LA-Pre	●	●	●	●	●
Premna angolensis Gürke	LA-Pre	●	FM200144	●	●	●
Premna barbata Wall. ex Schauer	LA-Pre	●	●	●	●	●
Premna bracteata Wall. ex C. B. Clarke	LA-Pre	●	●	●	●	●
Premna herbacea Roxb.	LA-Pre	●	●	●	●	●
Premna interrupta Wall. ex Schauer	LA-Pre	●	●	●	●	●
Premna ligustroides Hemsl.	LA-Pre	●	●	●	●	●
Premna menglaensis B. Li	LA-Pre	●	●	●	●	●
Premna microphylla Turcz.	LA-Pre	HQ427331	●	●	●	●

Premna nana Collett & Hemsl.	LA-Pre	●	●	●	●	●
**Premna obtusifolia* R. Br.	LA-Pre	-	●	FJ976163	-	●
Premna odorata Blanco	LA-Pre	HQ384494	HQ384812	HQ384866	HQ385142	HQ412925
Premna puberula Pamp.	LA-Pre	●	JQ322519	JQ322526	●	●
Premna punctulata C. B. Clarke	LA-Pre	●	●	●	●	●
Premna punicea C. Y. Wu	LA-Pre	●	●	●	●	●
Premna resinosa (Hochst.) Schauer	LA-Pre	-	●	●	-	●
Premna szemaoensis C. Pei	LA-Pre	●	●	●	●	●
Premna tanganyikensis Moldenke	LA-Pre	-	●	●	-	●
Premna tapintzeana Dop	LA-Pre	●	●	●	●	●
Premna tenii C. Pei	LA-Pre	●	●	●	●	●
Prostanthera calycina F. Muell. ex Benth.	LA-Pro	HQ384497	HQ384814	HQ384867	HQ385144	HQ412927
Prostanthera nivea A. Cunn. ex Benth.	LA-Pro	-	●	Z37430	AJ505403	●
Prostanthera rotundifolia R.Br.	LA-Pro	FM163283	U787021	L14008	-	●
**Prunella vulgaris* L.	LA-Ne	FJ395426	-	Z37433	-	DQ667508
Pseudocarpidium ilicifolium (A. Rich.) Millsp.	LA-Vi	-	●	-	-	●
**Pseudocarpidium wrightii* Millsp.	LA-Vi	-	-	-	-	JQ669063
**Pseudocaryopteris paniculata* (C. B. Clarke) P. D. Cantino	LA-Aj	AF477758	AY115894	-	-	-
**Puntia stenocaulis* Hedge	LA-Ne	-	-	-	AJ505424	AJ505545
**Pycnanthemum tenuifolium* Schrad.	LA-Ne	GU381750	-	-	-	GU381589
Pycnostachys reticulata (E. Mey.) Benth.	LA-Ne	JF270910	-	JF265567	AJ505395	AJ505516
Rosmarinus officinalis L.	LA-Ne	FR719113	-	Z37435	AJ505425	AY570465
Rostrinucula dependens (Rehder) Kudô	LA-La	HQ911405	●	-	FJ854074	FJ854302, FJ854189
Rotheca Raf.	LA-Aj	JX517676 *R. myricoides* (Hochst.) Steane & Mabb.	L49158 *R. incisa* (Klotzsch) Steane & Mabb.	U78713 *R. myricoides* (Hochst.) Steane & Mabb.	-	FJ952081 *R. myricoides* (Hochst.) Steane & Mabb.
Rubiteucris palmata (Benth. ex Hook. f.) Kudô	LA-Aj	AF477764	AY115883	-	-	JN575438

**Saccocalyx saturejoides* Coss. & Durand	LA-Ne	GU381796	-	-	-	GU381641
Salvia miltiorrhiza Bunge	LA-Ne	JX312195	JX312195	JX312195	JX312195	JX312195
**Satureja montana* L.	LA-Ne	AY840167	-	Z37455	-	AY840179
**Schizonepeta tenuifolia* (Benth.) Briq.	LA-Ne	-	-	FJ513155	-	EU186386
**Schnabelia* Hand.-Mazz.	LA-Aj	AF315296 *S. oligophylla* Hand.-Mazz.	AY115893 *S. nepetifolia* (Benth.) P. D. Cantino	-	-	-
Scutellaria L.	LA-Sc	HQ911383 *S. indica* L.	JQ322522 *S. indica* L.	Z37457 *S. alpina* L.	EU138289 *S. hirta* Sm.	EF546928, EF546848 *S. hirta* Sm.
Sideritis syriaca L.	LA-La	HQ911543	-	AF501999	FJ854082	FJ854304, FJ854198
**Spartothamnella teucriiflora* (F. Muell.) Moldenke	LA-Aj	-	AY310133	-	-	●
**Sphenodesme mollis* Craib	LA-Sy	-	FM200155	-	-	-
Sphenodesme sp.	LA-Sy	●	●	●	●	●
**Stachyopsis oblongata* (Schrenk) Popov & Vved.	LA-La	HQ911463	-	-	HQ911616	HQ911686, HQ911757
Stachys cretica L.	LA-La	HQ911554	-	HQ902776	FJ854100	FJ854316, FJ854215
Stenogyne Benth.	LA-La	HQ911567 *S. sessilis* Benth.	-	AF502024 *S. microphylla* Benth.	HQ911636 *S. sessilis* Benth.	HQ911705, HQ911776 *S. sessilis* Benth.
**Suzukia* Kudô	LA-La	HQ911562 *S. shikikunensis* Kudô	-	-	KF529765 *T. luchuensis* Kudô	FJ854331, FJ854237 *T. luchuensis* Kudô
Symphorema sp.	LA-Sy	●	●	●	●	●
**Synandra hispidula* (Michx.) Baill.	LA-La	HQ911427	-	-	HQ911597	HQ911670, HQ911737
**Syncolostemon rotundifolius* E. Mey. ex Benth.	LA-Ne	JF357832	-	-	AJ505402	AJ505523
Tectona grandis L. 1	LA	●	●	●	●	●
Tectona grandis L. 2	LA	●	●	●	●	●
Tectona grandis L. 3	LA	●	●	●	●	●
Tectona grandis L. 4	LA	●	●	●	●	●
Tectona grandis L. 5	LA	NC_020098	NC_020098	NC_020098	NC_020098	NC_020098
Tectona grandis L. 6	LA	HF567869	HF567869	HF567869	HF567869	HF567869
**Teijsmanniodendron bogoriense* Koord.	LA-Vi	-	FM200148	JF738613	-	-
**Teijsmanniodendron hollrungii*	LA-Vi	-	●	-	-	●

(Warb.) Kosterm.						
**Teijsmanniodendron pteropodum* (Miq.) Bakh.	LA-Vi	-	FM200149	-	-	-
**Teijsmanniodendron simplicifolium* Merr.	LA-Vi	-	FM200150	-	-	-
Tetraclea coulteri A. Gray	LA-Aj	-	AF130147	U78714	JN686533	FJ952068
Tetradenia Benth.	LA-Ne	JF270969 *T. riparia* (Hochst.) Codd	-	JF265627 *T. riparia* (Hochst.) Codd	AJ505399 *T. nervosa* Codd	AJ505520 *T. nervosa* Codd
Teucridium parvifolium Hook. f.	LA-Aj	-	U78684	U78715	-	JN408674
Teucrium L.	LA-Aj	HQ911378 *T. scorodonia* L.	AY115885 *T. canadense* L.	Z37468 *T. scorodonia* L.	FJ853996 *T. scorodonia* L.	JF694847 *T. scorodonia* L.
**Thorncroftia media* Codd	LA-Ne	-	-	-	AJ505400	AJ505522
**Thymbra spicata* L.	LA-Ne	GU381788	-	HQ902798	-	GU381632
Thymus L.	LA-Ne	AY840173 *T. serpyllum* L.	-	Z37472 *T. vulgaris* L.	AJ505423 *T. serpyllum* L.	AY570502 *T. serpyllum* L.
**Thuspeinanta persica* (Boiss.) Briq.	LA-La	HQ911550	-	-	FJ854126	FJ854334, FJ854240
Tinnea Kotschy & Peyr.	LA-Sc	HQ911385 *T. rhodesiana* S. Moore	U78709 *T. zambesiaca* Baker	U28886 *T. zambesiaca* Baker	HQ911582 *T. rhodesiana* S. Moore	HQ911652, HQ911721 *T. rhodesiana* S. Moore
Trichostema dichotomum L.	LA-Aj	AF336235	U78682	U28887	-	●
Vitex agnus-castus L. 1	LA-Vi	●	●	●	●	●
Vitex agnus-castus L. 2	LA-Vi	AB284182	U78707	U78716	HQ385143	HQ412926
Vitex axillariflora (Merr.) Bramley	LA-Vi	●	FM200151	●	●	●
Vitex buchananii Baker ex Gürke	LA-Vi	JX517569	●	JX573083	-	●
**Vitex glabrata R.Br.*	LA-Vi	AB284178	FM200159	-	-	-
**Vitex guanahacabibensis Borhidi*	LA-Vi	-	●	-	-	●
**Vitex lignum-vitae A. Cunn. ex Schauer*	LA-Vi	-	●	-	-	●
**Vitex megapotamica (Spreng.) Moldenke*	LA-Vi	-	●	-	-	-
Vitex negundo L.	LA-Vi	AB284176	JQ322520	JQ322525	-	DQ304786
Vitex pinnata L.	LA-Vi	AF315305	FM200169	●	-	-
Vitex queenslandica (Munir)	LA-Vi	●	FM200177	●	●	●

Bramley						
Vitex thailandica Bramley	LA-Vi	-	FM200141	●	●	●
Vitex trifolia L.	LA-Vi	AB284175	FM200171	GQ436526	AJ505416	AJ505539
Vitex trifolia subsp. litoralis Steenis	LA-Vi	AB284177	●	-	-	●
Vitex turczaninowii Merr.	LA-Vi	●	FM200176	●	●	●
**Warnockia scutellarioides* (Engelm. & A. Gray) M. W. Turner	LA-La	HQ911430	-	-	HQ911599	EF546971, EF546895
Wenchengia alternifolia C. Y. Wu & S. Chow	LA-Sc	●	JQ322523	JQ322528	JX893281	JX893310, JX893339
Westringia Sm.	LA-Pro	HQ911373 *W. rigida* R.Br.	GQ381197 *W. rigida* R.Br.	Z37474 *W. rosmariniformis* Sm.	HQ911569 *W. rigida* R.Br.	HQ911707 *W. rigida* R.Br.
**Zataria multiflora Boiss.*	LA-Ne	GU381782	-	-	-	GU381626
**Ziziphora taurica M. Bieb.*	LA-Ne	GU381673	-	HQ902813	-	DQ667501

Supplementary Table S2. Information about PCR amplification primers for matK, ndhF, rbcL, rps16 and trnL-F regions.

Markers	Primer name	Sequences (5′→3′)	References
matK	323f	ATTNTCAAATCNTAKCAGAGGGG	Andersson[3]
	1189r	TACNATCAATTCATTCAATATTYCC	Andersson[3]
ndhF	ndh972F	GTCTCAATTGGGTTATATGATG	Olmstead and Sweere[4]
	ndh#2112R	CCCYASATATTTGATACCTTCKCC	Olmstead and Reeves[5]
rbcL	rbcL aF	ATGTCACCACAAACAGAGACT AAAGC	Hasebe *et al.*[6]
	rbcL cR	GCAGCAGCTAGTTCCGGGCTCCA	Hasebe *et al.*[6]
rps16	rpsF	GTGGTAGAAAGCAACGTGCGACTT	Oxelman *et al.*[7]
	rpsR2R	TCGGGATCGAACATCAATTGCAAC	Oxelman *et al.*[7]
trnL-F	c	CGAAATCGGTAGACGCTACG	Taberlet *et al.*[8]
	f	ATTTGAACTGGTGACACGAG	Taberlet *et al.*[8]

Supplementary Figures

Fig. S1. Strict consensus tree resulted from MP analysis based on *matK* sequence with gaps treated as simple indels (L = 2384 CI = 0. 53 RI = 0. 87). The topology of the optimal ML tree was congruent with the MP tree. Numbers above branches indicated bootstrap support values ≥ 50% in MP and ML analysis, respectively. Multiple accessions of the same species are numbered according to Supplementary Table S1. Subfamilies and tribes recognized by Olmstead[2] were covered by gray boxes, and genera without certain sabfamilial placement at present were marked in red font.

Fig. S2. Strict consensus tree resulted from MP analysis based on *ndhF* sequence with gaps treated as simple indels (L = 3891 CI = 0. 43 RI = 0. 75). Notations as in Fig. S1.

Fig. S3. Strict consensus tree resulted from MP analysis based on *rbcL* sequence with gaps treated as simple indels (L = 1445 CI = 0. 32 RI = 0. 73). Notations as in Fig. S1.

Fig. S4. Strict consensus tree resulted from MP analysis based on *rps16* sequence with gaps treated as simple indels (L = 1757 CI = 0. 51 RI = 0. 84). Notations as in Fig. S1.

Fig. S5. Strict consensus tree resulted from MP analysis based on *trnL-F* sequence with gaps treated as simple indels (L = 1662 CI = 0. 47 RI = 0. 86). Notations as in Fig. S1.

Fig. S6. MP strict consensus tree from the analysis of the combined cpDNA (*matK* + *ndhF* + *rbcL* + *rps16* + *trnL-F*) dataset D270 with gaps treated as simple indels (L = 11084 CI = 0. 47 RI = 0. 83). Bootstrap support values ≥ 50% are marked above branches. Multiple accessions of the same species are numbered according to Supplementary Table S1. A single generic name represented that the combined sequences pooled from different species of the genus. Subfamilies and tribes recognized by Olmstead[2] were covered by gray boxes with different grey level, while new subfamilies (Cymarioideae, Peronematoideae, and Premnoideae) and clades (*Callicarpa*, and *Tectona*) proposed in this study were covered by pink boxes and marked in red bold font.

Fig. S7. Optimal ML tree from the analysis of the combined cpDNA (*matK* + *ndhF* + *rbcL* + *rps16* + *trnL-F*) dataset D270 with gaps treated as simple indels. Bootstrap support values ≥ 50% are marked above branches. Notations as in Fig. S6.

Fig. S8. 50% majority-rule consensus tree from the BI analysis of the combined cpDNA (*matK* +

ndhF + *rbcL* + *rps16* + *trnL-F*) dataset D270 with gaps treated as simple indels. Posterior probabilities values ≥ 0. 90 are marked above branches. Notations as in Fig. S6.

Fig. S9. MP strict consensus tree from the analysis of the combined cpDNA (*matK* + *ndhF* + *rbcL* + *rps16* + *trnL-F*) dataset D270 with gaps treated as missing data. Bootstrap support values ≥ 50% are marked above branches. Notations as in Fig. S6.

Fig. S10. Optimal ML tree from the analysis of the combined cpDNA (*matK* + *ndhF* + *rbcL* + *rps16* + *trnL-F*) dataset D270 with gaps treated as missing data. Bootstrap support values ≥ 50% are marked above branches. Notations as in Fig. S6.

Fig. S11. 50% majority-rule consensus tree from the BI analysis of the combined cpDNA (*matK* + *ndhF* + *rbcL* + *rps16* + *trnL-F*) dataset D270 with gaps treated as missing data. Posterior probabilities values ≥ 0. 90 are marked above branches. Notations as in Fig. S6.

Fig. S12. The Bayesian 50% majority-rule consensus phylogram based on combined cpDNA (*matK* + *ndhF* + *rbcL* + *rps16* + *trnL-F*) dataset D155 with gaps treated as missing data. The topology of the ML and MP trees were congruent with the BI tree. Support values displayed on the branches follow the order BI-PP/ML-BS/MP-BS ("-" indicates support values of less than 0. 90 in BI or 50% in ML and MP analyses, respectively). The bold lines indicate the three support values get full scores simultaneously. Multiple accessions of the same species are numbered according to Supplementary Table S1. A single generic name represented that the combined sequences pooled from different species of the genus. Subfamilies and tribes recognized by Olmstead[2] were covered by gray boxes with different grey level, while new subfamilies (Cymarioideae, Peronematoideae, and Premnoideae) and clades (*Callicarpa*, and *Tectona*) proposed in this study were covered by pink boxes and marked in red bold font.

References

1. Harley, R. M. *et al.* in *Families andgenera of vascular plants*, Vol. 7 (eds Kubitzki, K. & Kadereit, J. W.), Labiatae, 167–275 (Springer, Berlin, 2004).
2. Olmstead, R. G. 2012. *A synoptical classification of the Lamiales, version 2.4* (2012) Available at: http://depts.washington.edu/phylo/Classification.pdf. (Accessed: 5th April 2013).
3. Andersson, S. On the phylogeny of the genus *Calceolaria* (Calceolariaceae) as inferred from ITS and plastid matK sequences. *Taxon* **55**, 125–137 (2006).

4. Olmstead, R. G. & Sweere, J. A. Combining data in phylogenetic systematics: an empirical approach using three molecular data sets in the Solanaceae. *Syst. Biol.* **43**, 467–481 (1994).

5. Olmstead, R. G. & Reeves, P. A. Evidence for the polyphyly of the Scrophulariaceae based on chloroplast *rbcL* and *ndhF* sequences. *Ann. Missouri Bot. Gard.* **82**, 176–193 (1995).

6. Hasebe, M., Omori, T. , Nakazawa, M., Sano, T., Kato, M. & Iwatsuki, K. *RbcL* gene sequences provide evidence for the evolutionary lineages of leptosporangiate ferns. *Proc. Nat. Acad. Sci.* **91**, 5730–5734 (1994).

7. Oxelman, B., Liden, M. & Berglund, D. Chloroplast *rps16* intron phylogeny of the tribe Sileneae (Caryophyllaceae). *Pl. Syst. Evol.* **206**, 393–410 (1997).

8. Taberlet, P., Gielly, L., Pautou, G. & Bouvet, J. 1991. Universal primers for amplification of three non-coding regions of chloroplast DNA. *Pl. Molec. Biol.* **17**, 1105–1109.

Supplementary Fig. S1

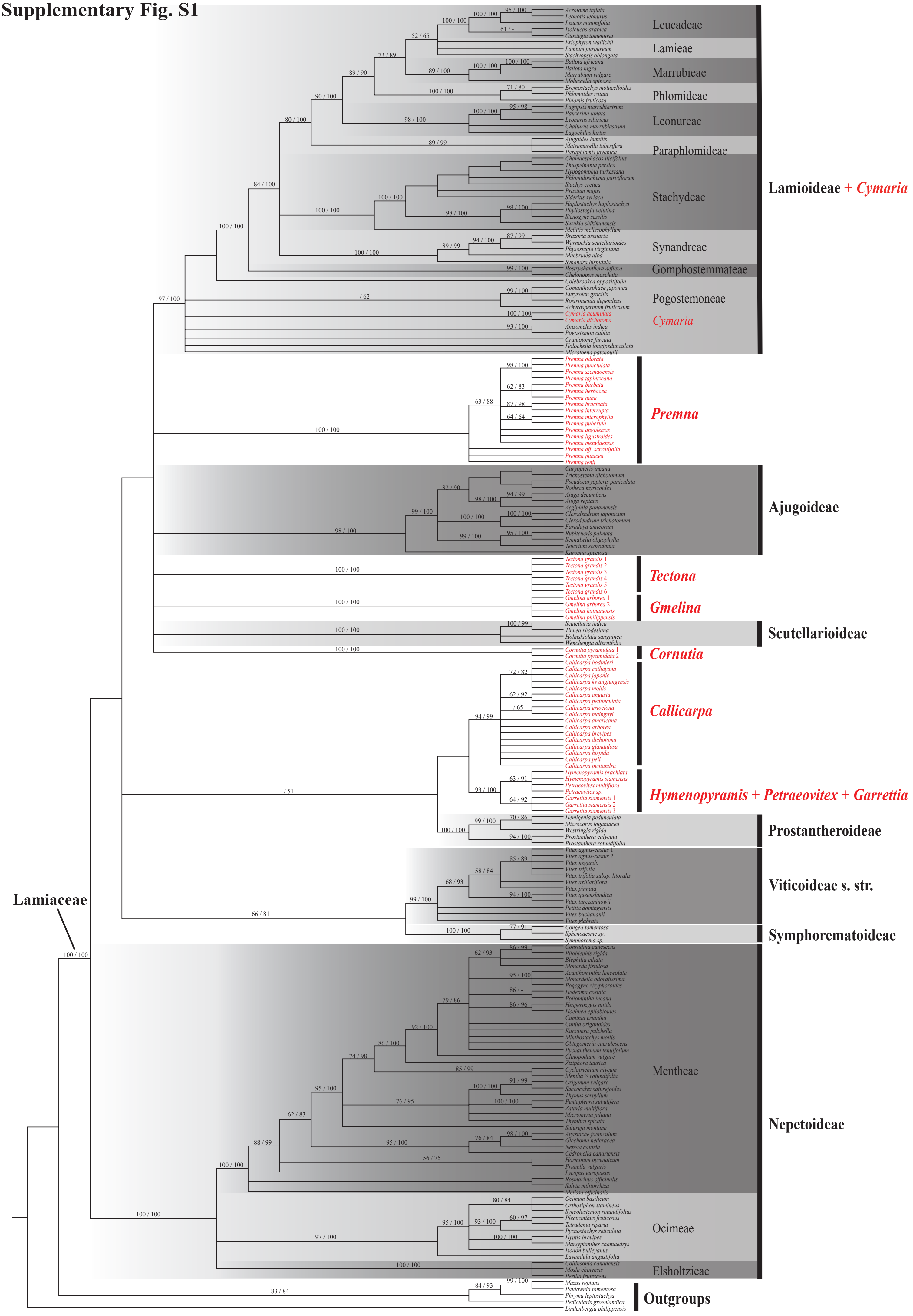

Supplementary Fig. S2

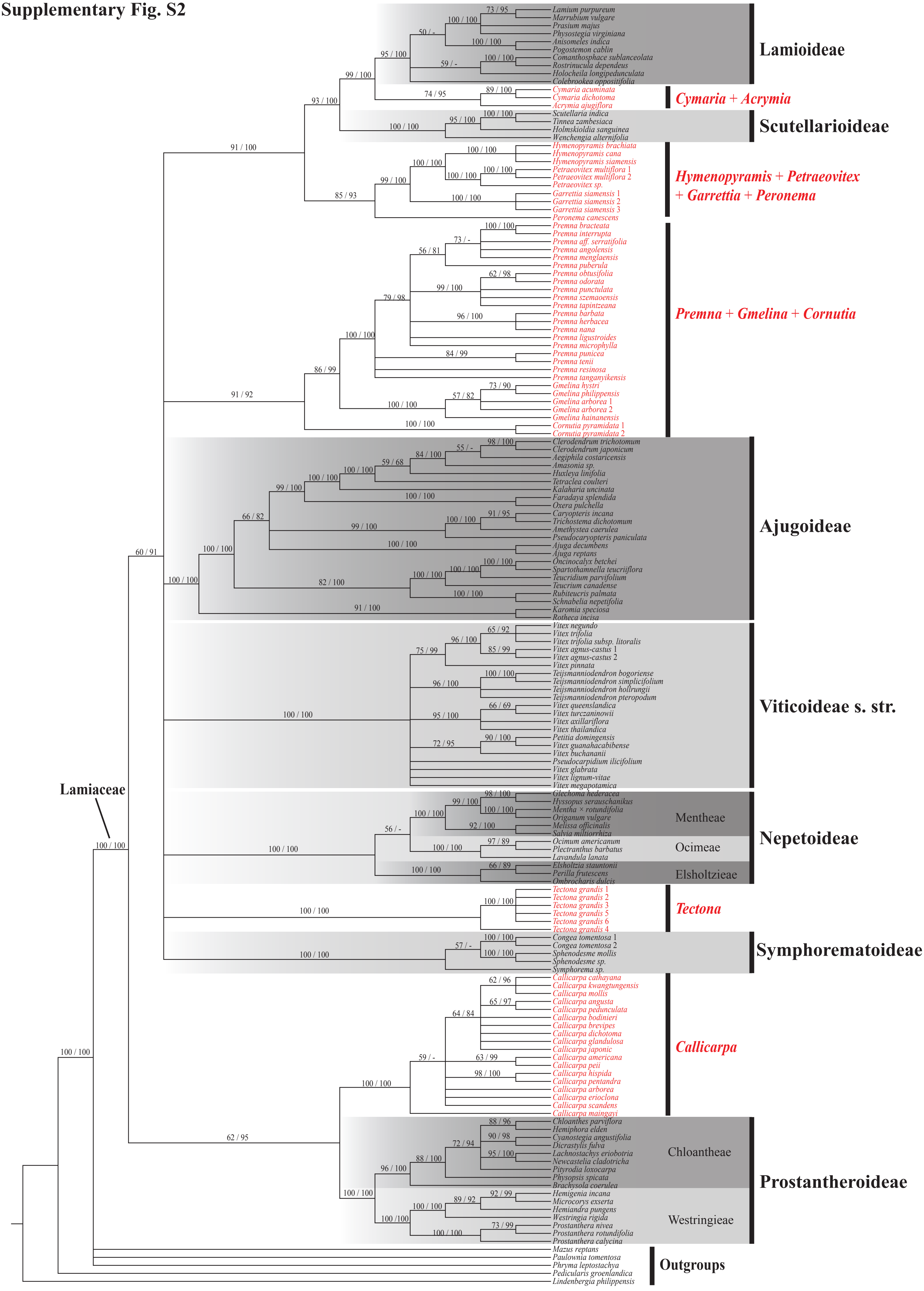

Supplementary Fig. S3

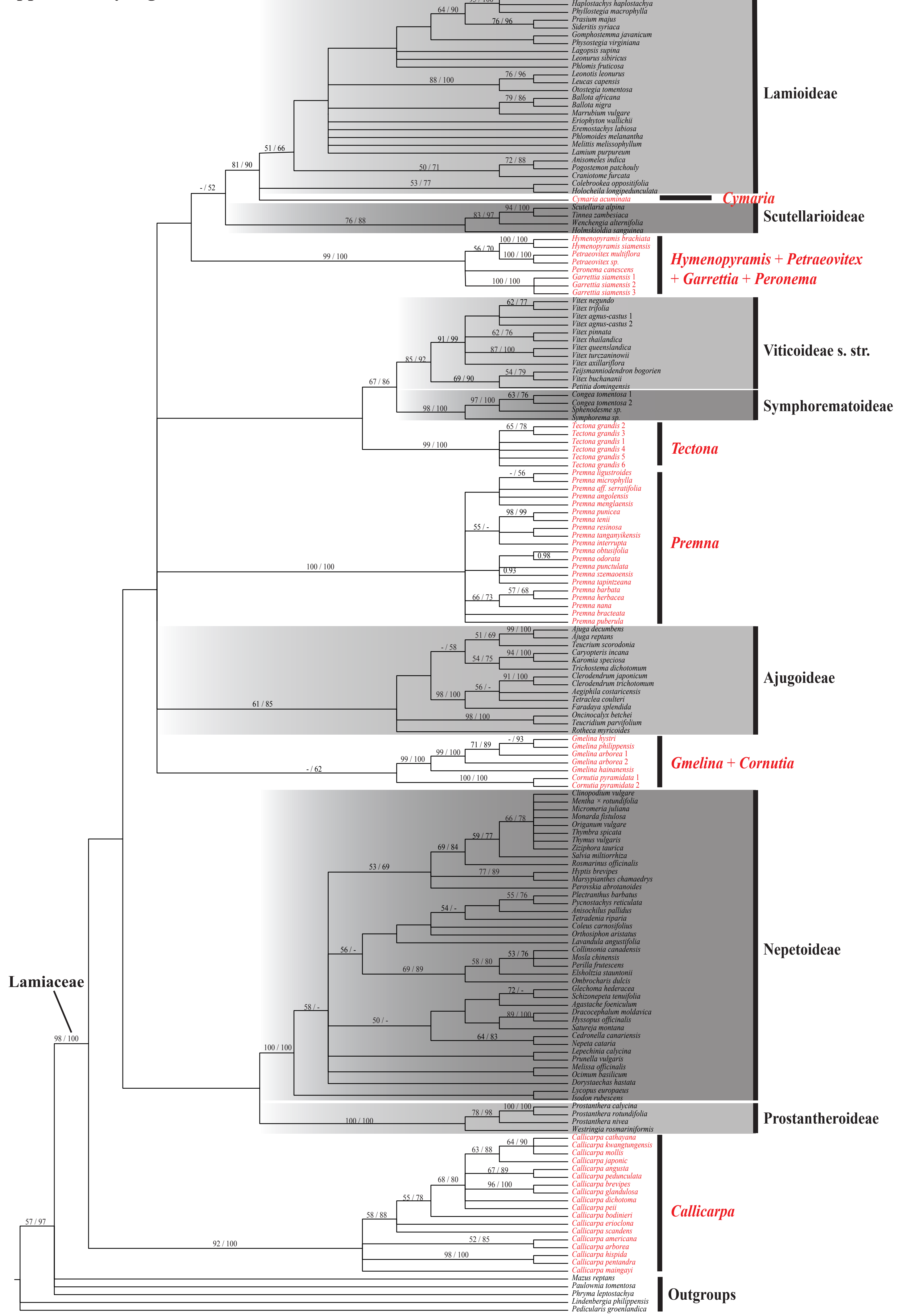

Supplementary Fig. S4

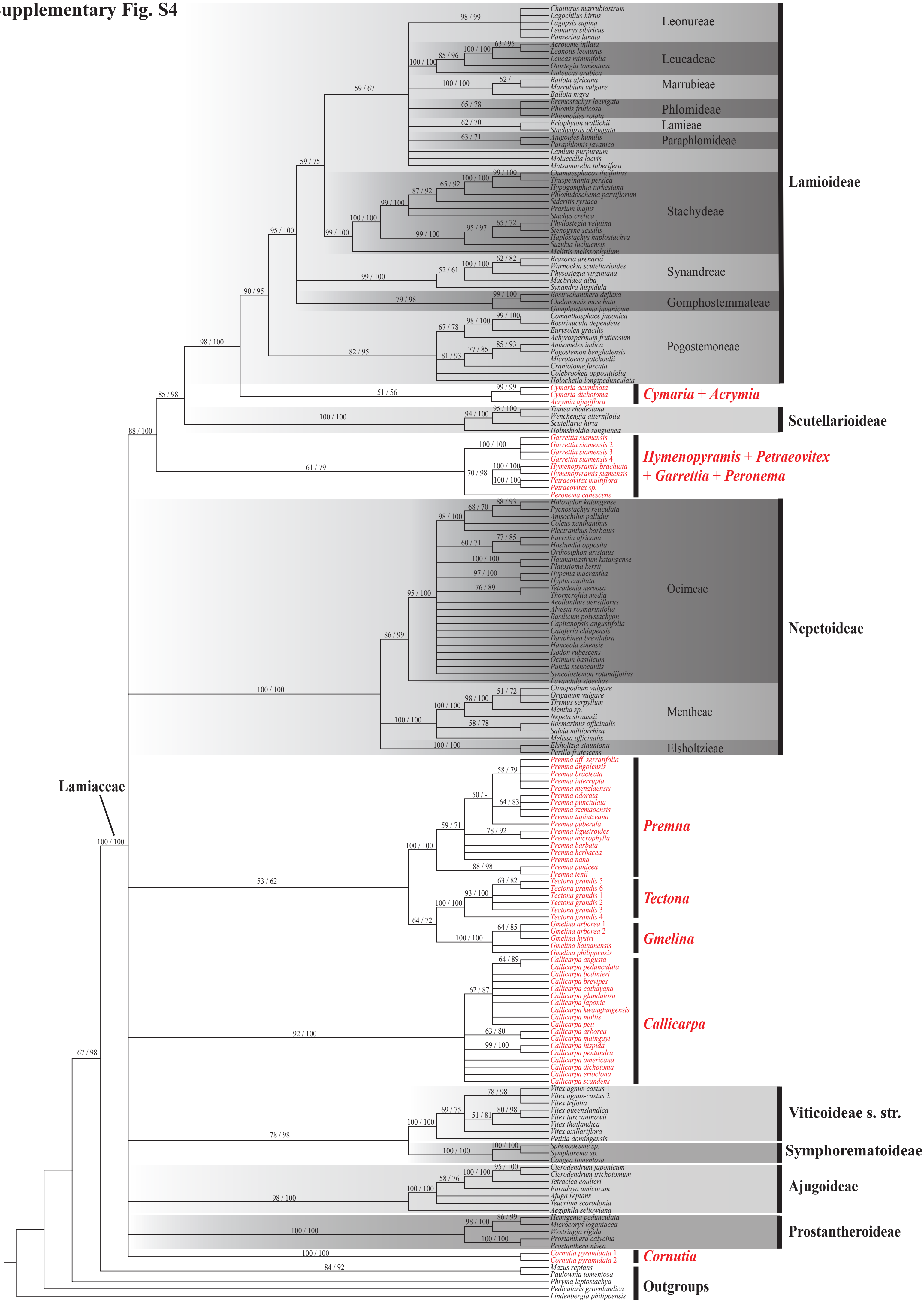

Supplementary Fig. S5

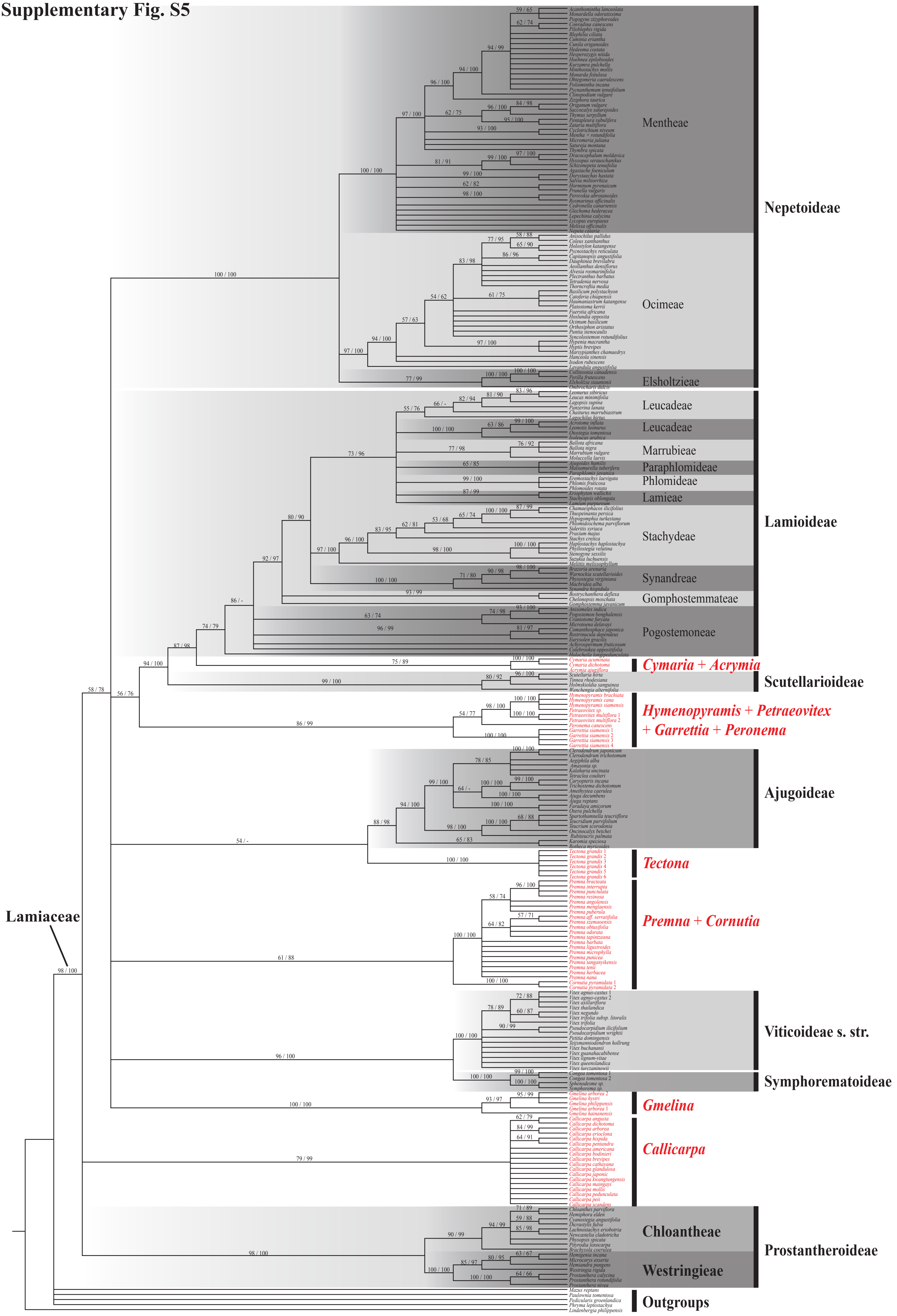

Supplementary Fig. S6

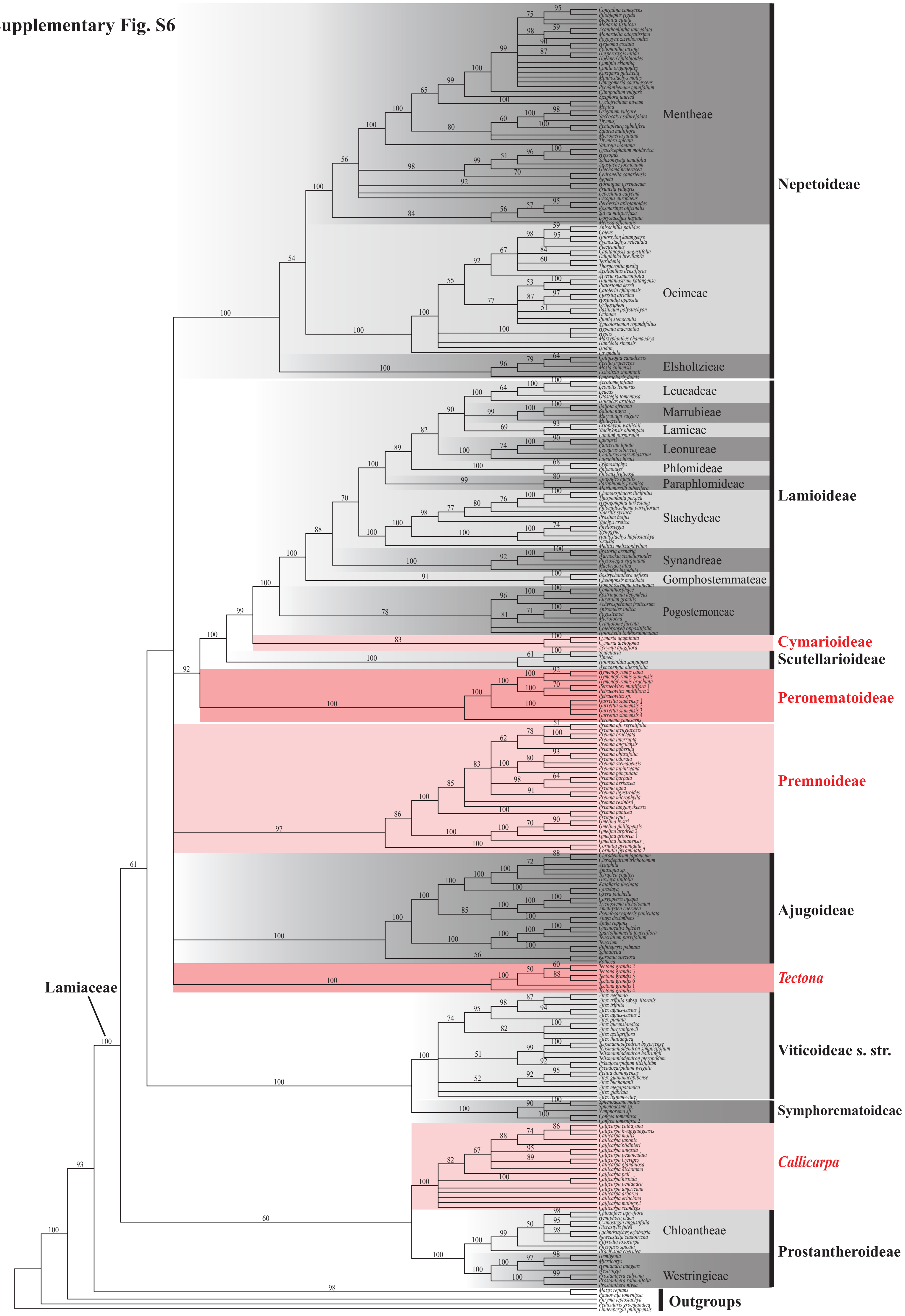

Supplementary Fig. S7

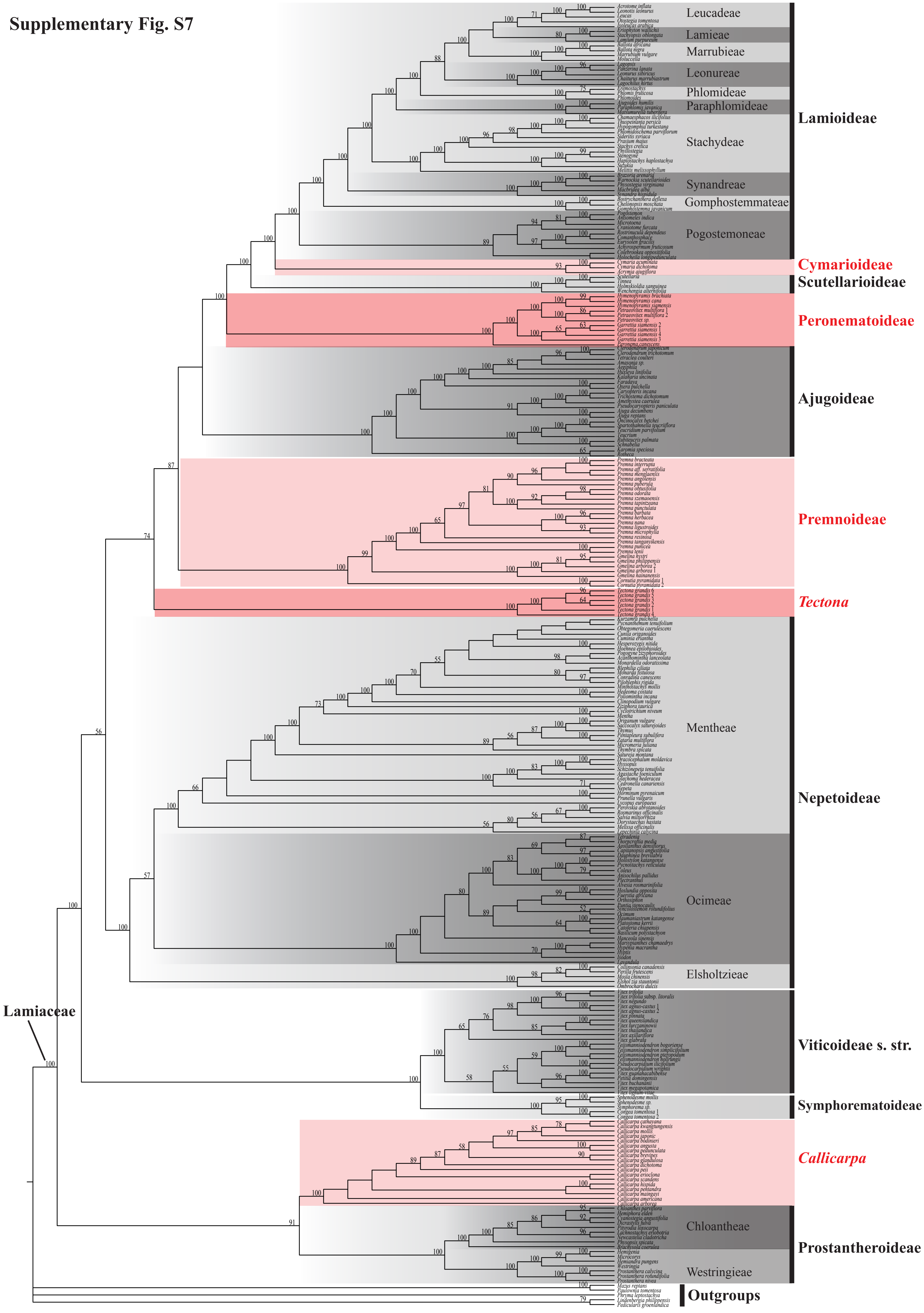

Supplementary Fig. S8

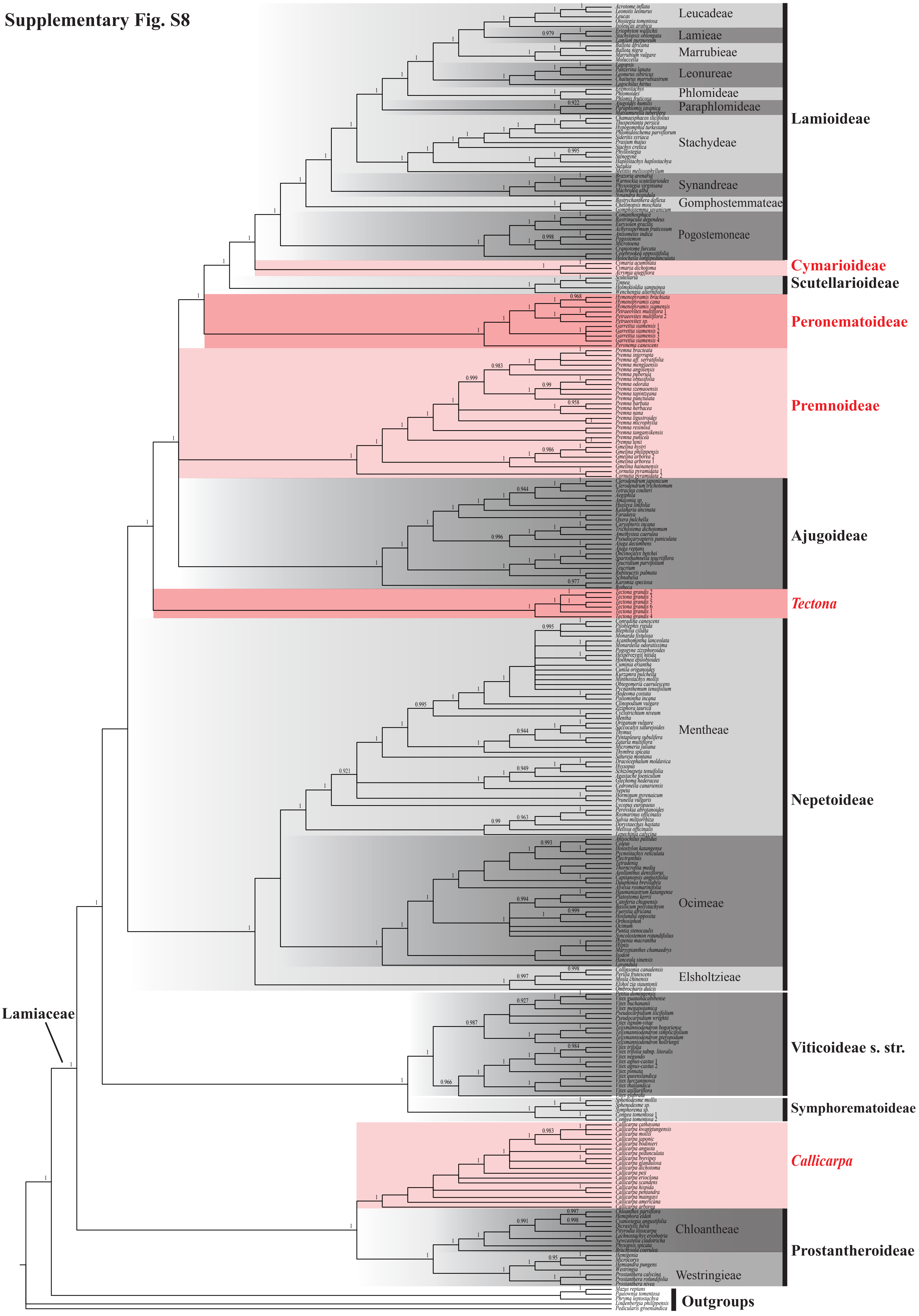

Supplementary Fig. S9

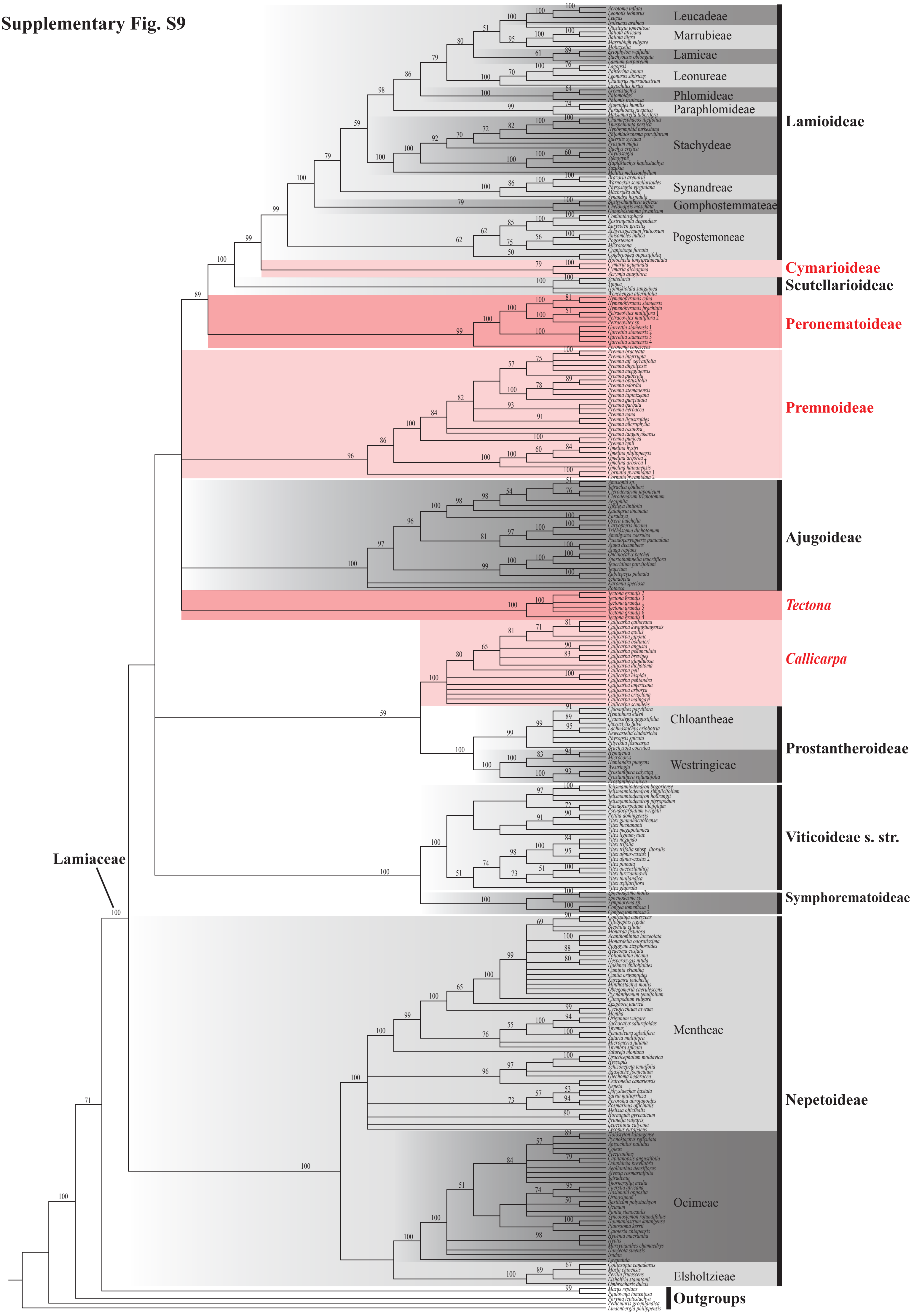

Supplementary Fig. S10

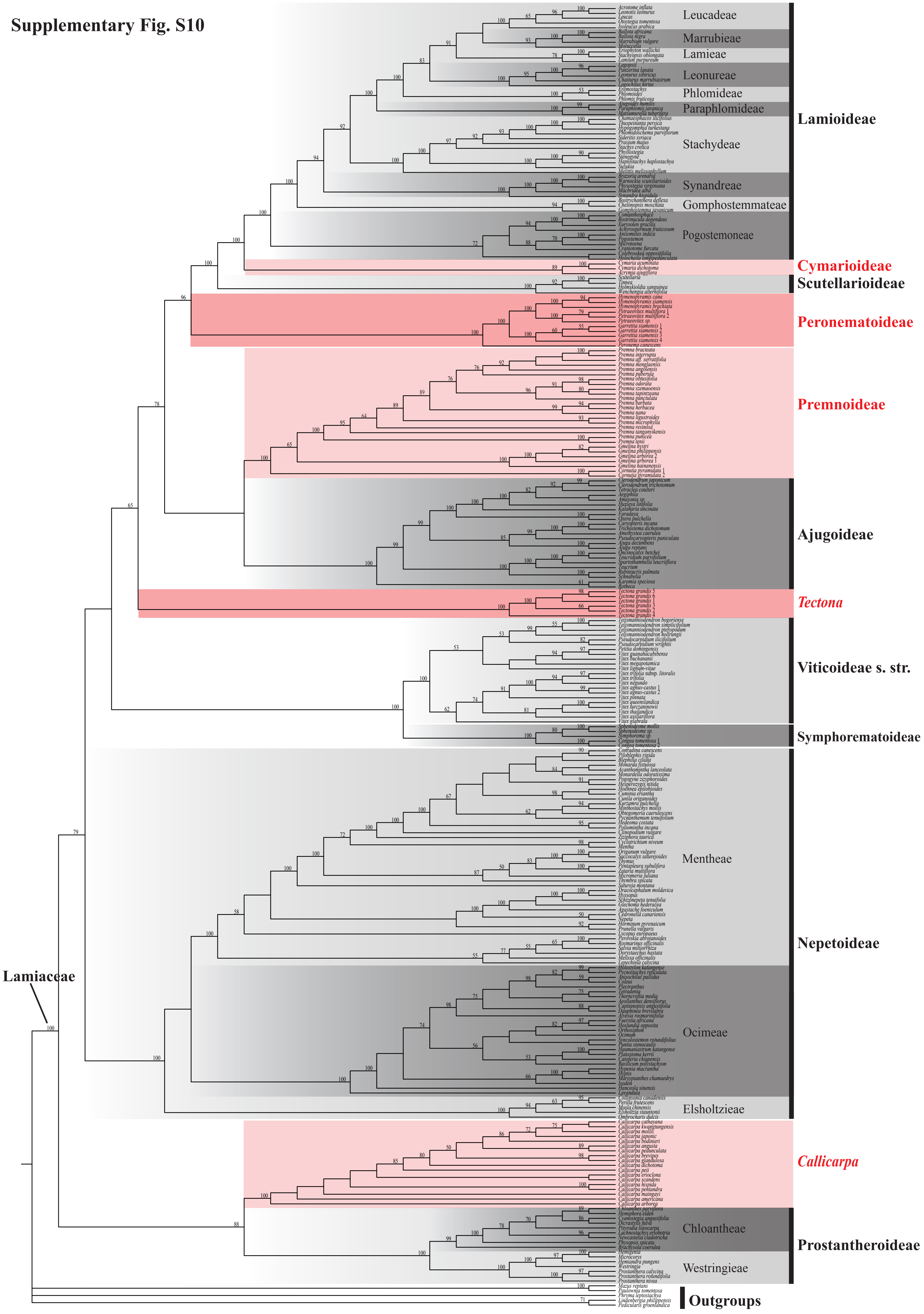

Supplementary Fig. S11

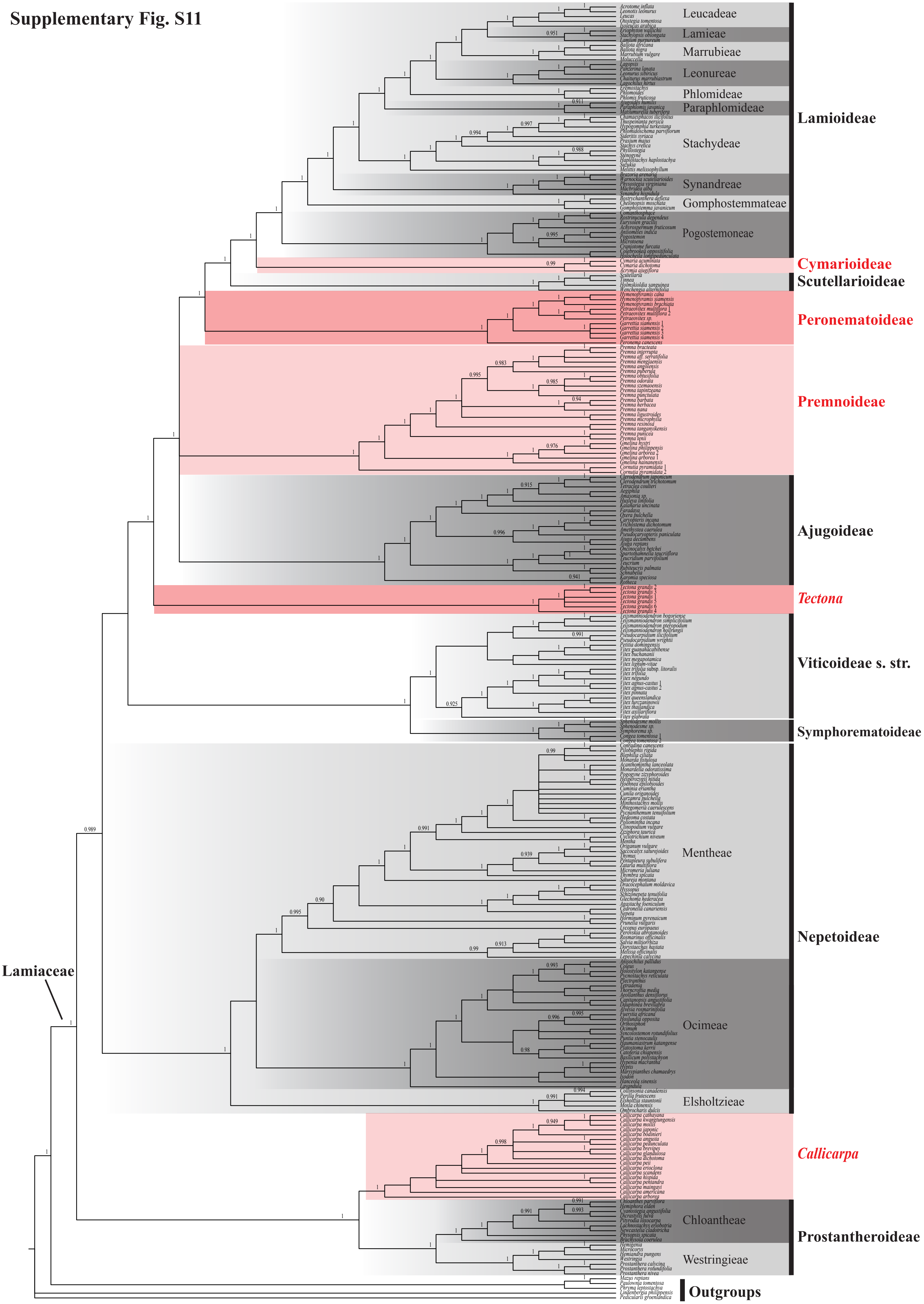

Supplementary Fig. S12

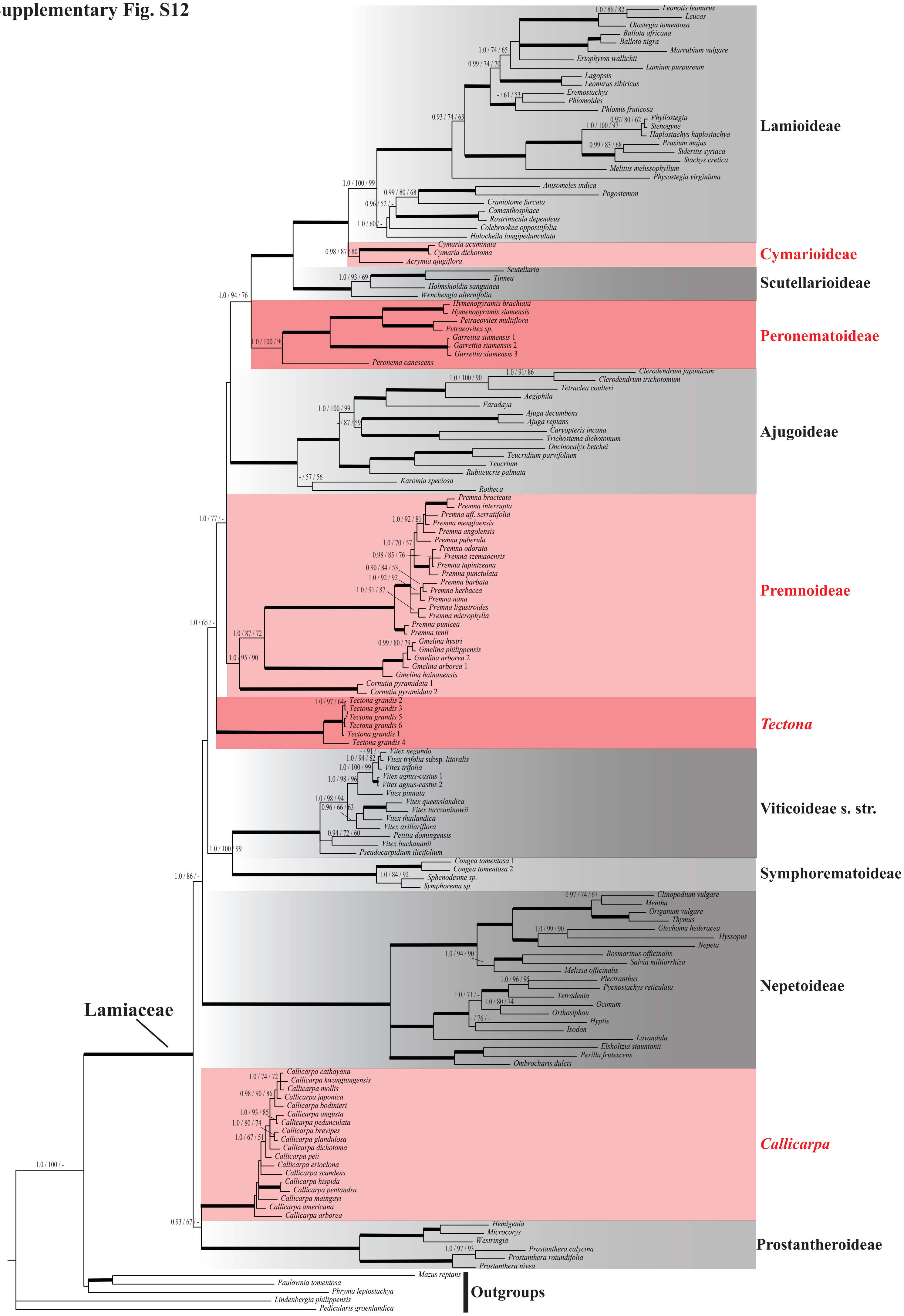

Supplement: Supplementary Information [file srep34343-s1.pdf]
